# Supplementary figures and images for: Analysis of localized cAMP perturbations within a tissue reveal the effects of a local, dynamic gap junction state on ERK signaling
Source: PLoS Comput Biol. 2022 Mar 30;18(3):e1009873. doi: 10.1371/journal.pcbi.1009873 (PMC9000136; doi:10.1371/journal.pcbi.1009873)

A

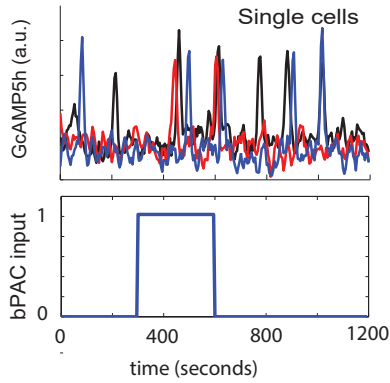

D

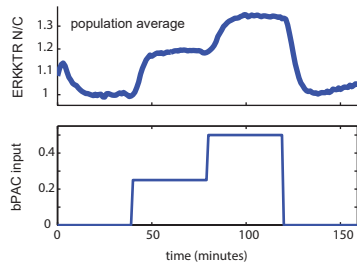

B

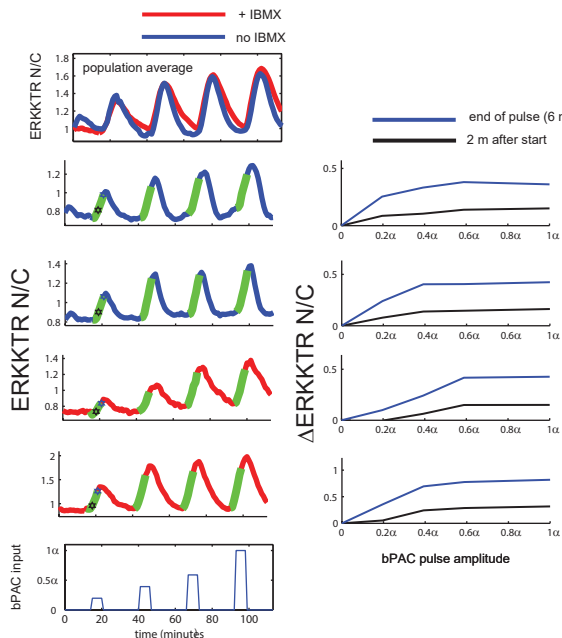

C

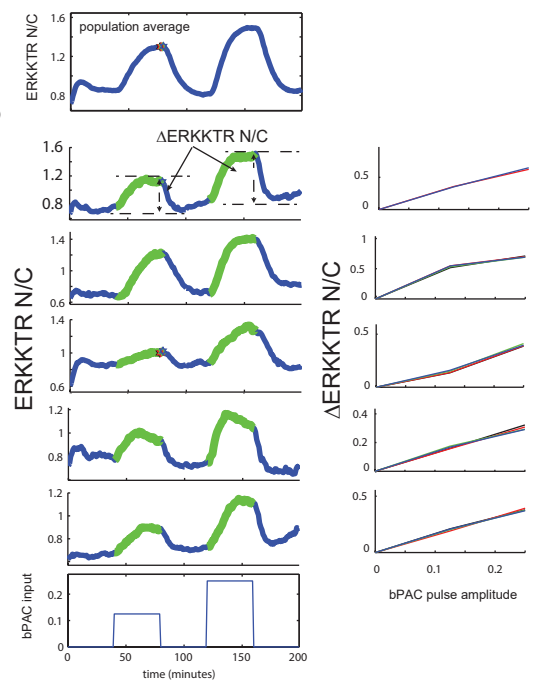

E

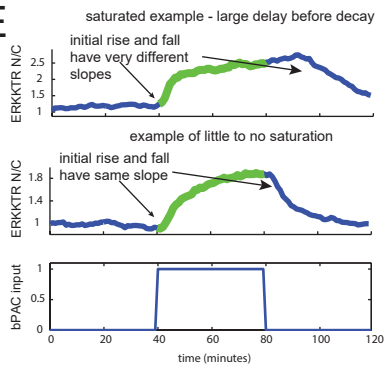

F

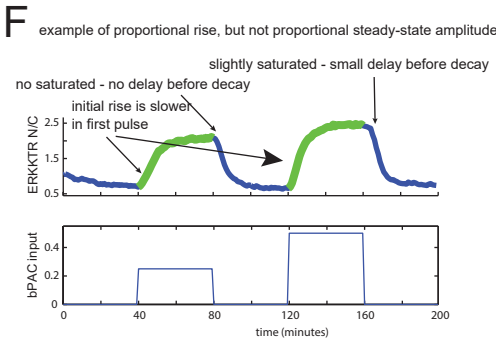

G

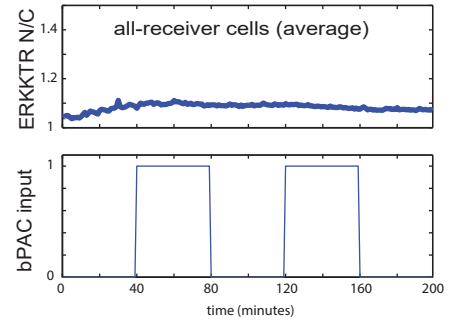

Supplement: S1 Fig — (A) Activation of bPAC results in higher firing rate of Ca2+ spikes in single cells. (B-C) ERK-KTR N/C signal due to bPAC input pules of increasing amplitude (maximum amplitude of one corresponds to the maximum blue light photon flux of the system with a neutral density filter). Left panel: Top plot: emitter population ERK-KTR N/C signal average. Middle plots: single cell ERK-KTR N/C signals. Green segments on the ERK-KTR N/C signal show when the bPAC pulse is on. Bottom plot: bPAC input pulse sequence. Right panel: ΔERK-KTR N/C versus bPAC pulse amplitude. ΔERK-KTR N/C is the difference between the ERK-KTR N/C signal at the start of the pulse and the signal at the specified time of measurement. (B) ERK-KTR N/C signal due to 6 minute bPAC input pulses with increasing amplitudes of .2α, .4α, .6α, and 1α for all-emitter monolayers for cells with phosphodiesterase (PDE) inhibition (IBMX, red) and with no IBMX (blue). For these experiments a neutral density filter was not used and α represents the resulting scaled increase in photon flux where α ≈ 7. ΔERK-KTR measurements are taken at 2 minutes and 6 minutes after the start of the pulse (see black (2m) and blue (6m) stars in first pulse of the top single cell plot, for example). (C) ERK-KTR N/C signal due to 40 minute bPAC input pulses with increasing amplitudes of amplitudes of .125 and .25. ΔERK-KTR measurements are taken at the end of each pulse (see top single cell plot). (D) ERK-KTR N/C signal due to 40 minute bPAC input steps with increasing amplitudes of .2 and .4 for an all-emitter monolayer. (E) Single cell exhibiting saturation in its ERK-KTR N/C signal (top plot) with a large delay before decaying after the bPAC input pulse (bottom plot) has shut off. Compare with a single cell that is within the dynamic range of the reporter (middle plot) and with a decay delay on the order of a minute or two, the natural delay of the reporter when changes in cAMP occur. (F) Example of single cell ERK-KTR N/C signal that exh [file pcbi.1009873.s001.pdf]

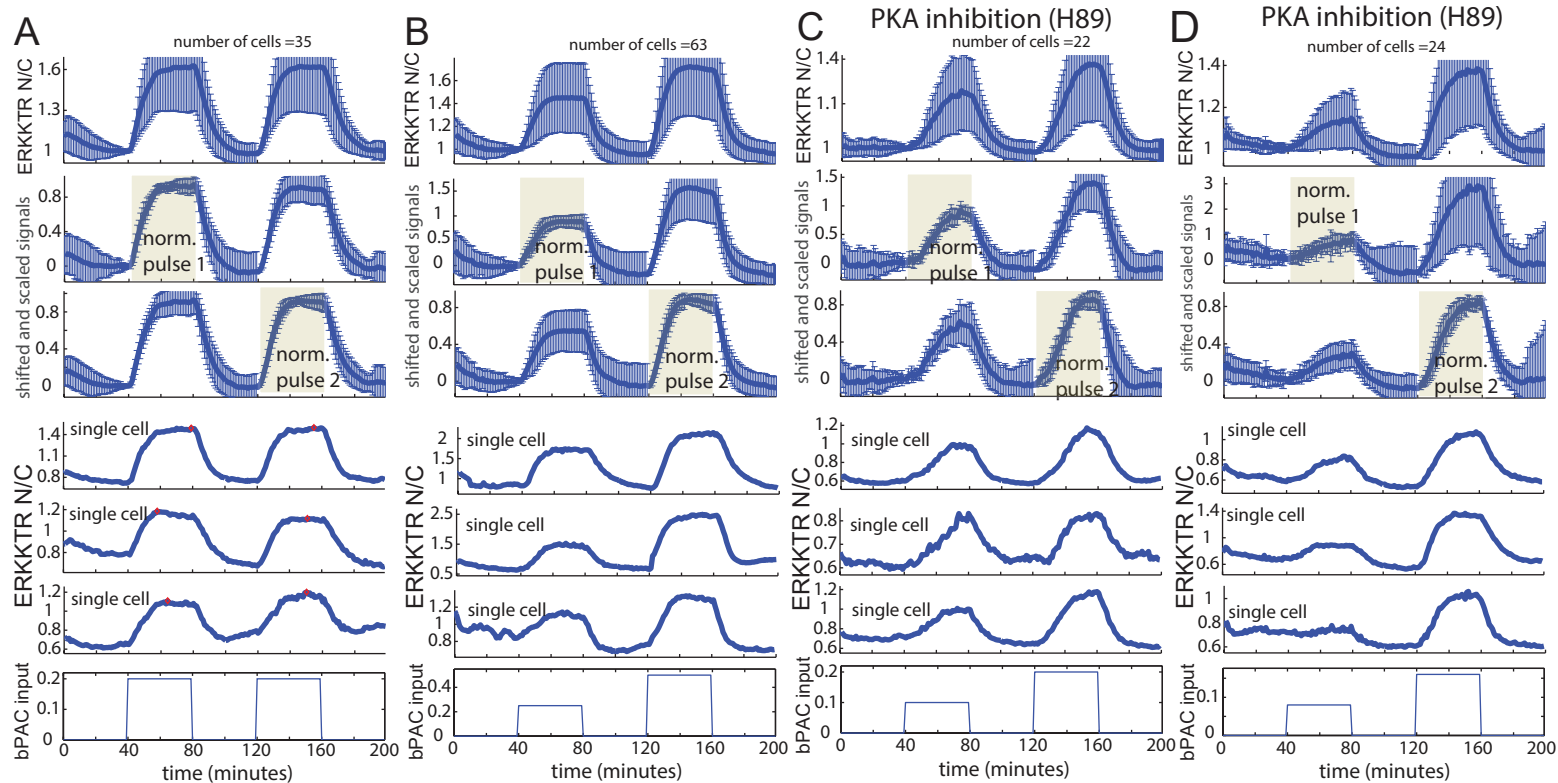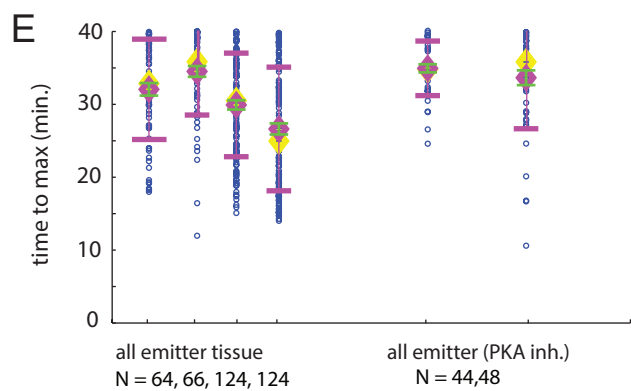

Supplement: S2 Fig — (A-D) Calculation of the population mean and standard deviation of the ERK-KTR N/C signal under different normalization approaches (same approach as in Fig 2E and 2F). Top 3 plots: For a given normalization approach, each single cell ERK-KTR N/C signal is shifted such that the signal at the beginning of the first pulse is zero and then normalized. Mean and standard deviation are calculated to just before the start of the second pulse. This process is the then repeated for the second pulse enforcing the standard deviation is zero at the beginning of each pulse. Top plot: mean ± standard deviation. Second plot from top: mean ± standard deviation calculated after each shifted signal is normalized by difference between the peak value of the signal during the first pulse and the value at the beginning of the first pulse. This removes the effects of peak amplitude variability in the first pulse. Third plot from top: same as prior normalization approach of the first pulse, but applied to the second pulse instead. This removes the effects of peak amplitude variability in the second pulse. Second, third, and fourth plots from the bottom: single cell ERK-KTR N/C signals. Bottom plot: bPAC input pulse sequence. (A) All-emitter experiment with identical bPAC input pulse amplitudes. (B) All-emitter experiment with increasing bPAC input pulse amplitudes. (C) All-emitter experiment with increasing bPAC input pulse amplitudes with PKA inhibition. (D) All-emitter experiment with increasing bPAC input pulse amplitudes with PKA inhibition. (E) Scatter plot of time-to-max times (same approach as described in Fig 3C) for single cell ERK-KTR N/C signals taken from all-emitter experiments from (A), (B), and Fig 2E and 2F, and all-emitter experiments with PKA inhibition from (C) and (D). Mean (magenta diamond) ± standard deviation (magenta error bars), error bars in the mean (green, standard deviation/N) representing 95 percent confidence interval in the mean, and median (yellow diamond) a [file pcbi.1009873.s002.pdf]

# A heterogeneity in emitter response for small emitter clusters and single emitter clusters

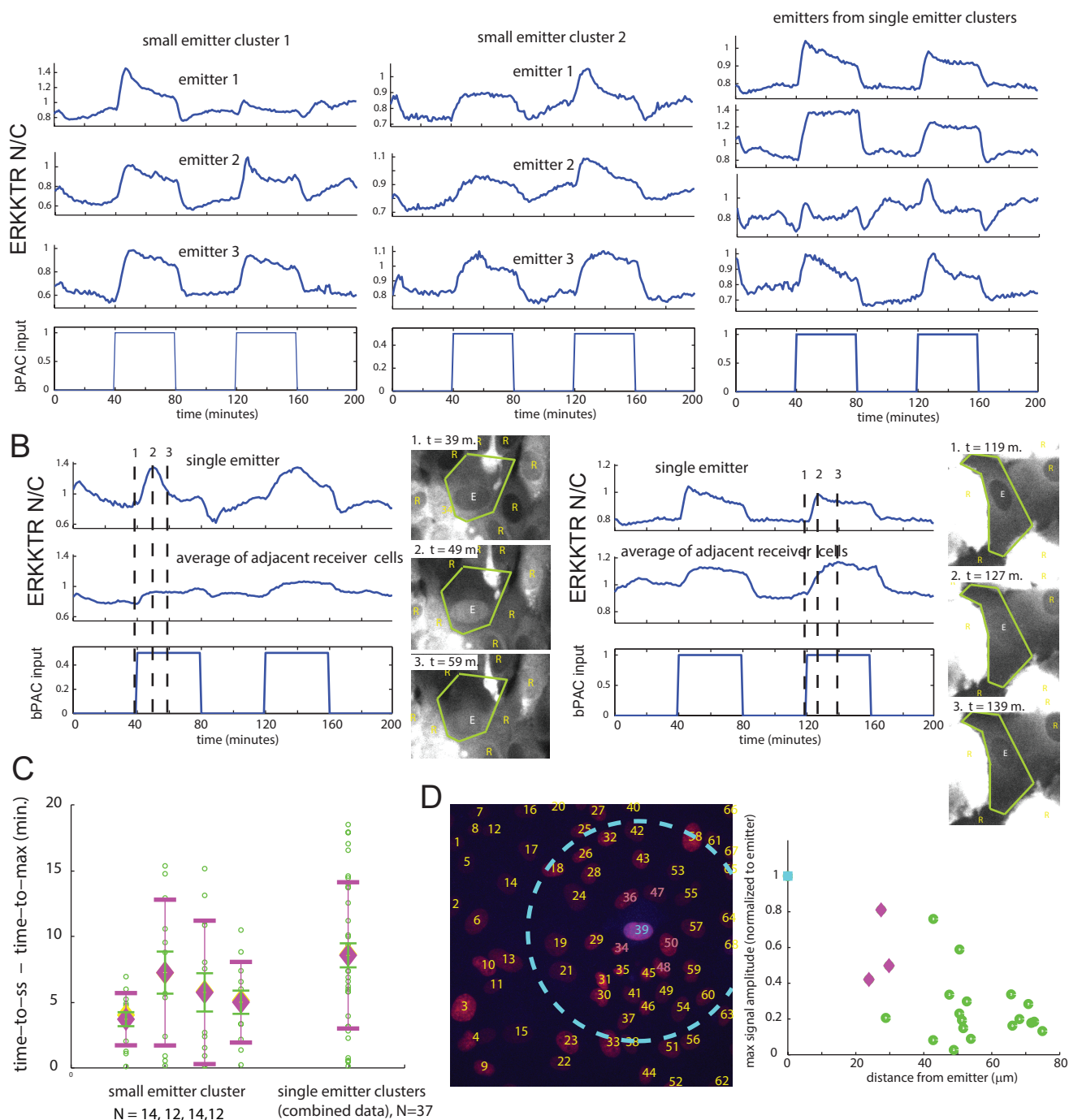

Supplement: S3 Fig — A) Heterogeneity is observed in emitter response for small emitter clusters and single emitter clusters. Left two panels: Example plots of emitter ERK-KTR N/C signals from two small emitter clusters of size N = 5 and 6, respectively. Bottom plot: bPAC input. Right panel: Examples of emitter ERK-KTR N/C signals from different single emitter clusters. Bottom plot: bPAC input. (B) Examples of single emitter and adjacent receiver average ERK-KTR N/C signals along with time snapshots of emitter ERK-KTR response (within outline) displaying the change in nuclear ERK-KTR after overshoot. (C) Scatter plot of time-to-ss—time-to-max statistics for the small-emitter-cluster experiments (green, with left most from experiment presented in Fig 3A), and combined data for 10 single-emitter-cluster experiments, all presented Fig 3D. Mean (magenta diamond) ± standard deviation (magenta error bars), error bars in the mean (green, standard deviation/N) representing 95 percent confidence interval in the mean, and median (yellow diamond) are also plotted. Number of data points N used are displayed below each group label. (D) Single emitter experiment that shows a general decreasing trend of signal amplitude vs. distance from the emitters. Left Panel: Image of single emitter (purple nucleus, cyan label), adjacent receivers (red nuclei, magenta label) and all other receivers (red nuclei, yellow label). Cyan circle centered at emitter represents a radius of 80 μm (.4 μm per pixel). Right Panel: Normalized steady-state signal amplitude (relative to emitter) of receiver cells as a function of distance from emitter (cyan square at zero distance). Steady-state signals are measured at the end of the bPAC input pulse. Adjacent receiver cells from image are labeled as magenta diamonds. All other receiver cells are labeled as green circles. Cells that did not respond to bPAC were not included (including adjacent receivers 34 and 48). (PDF) [file pcbi.1009873.s003.pdf]

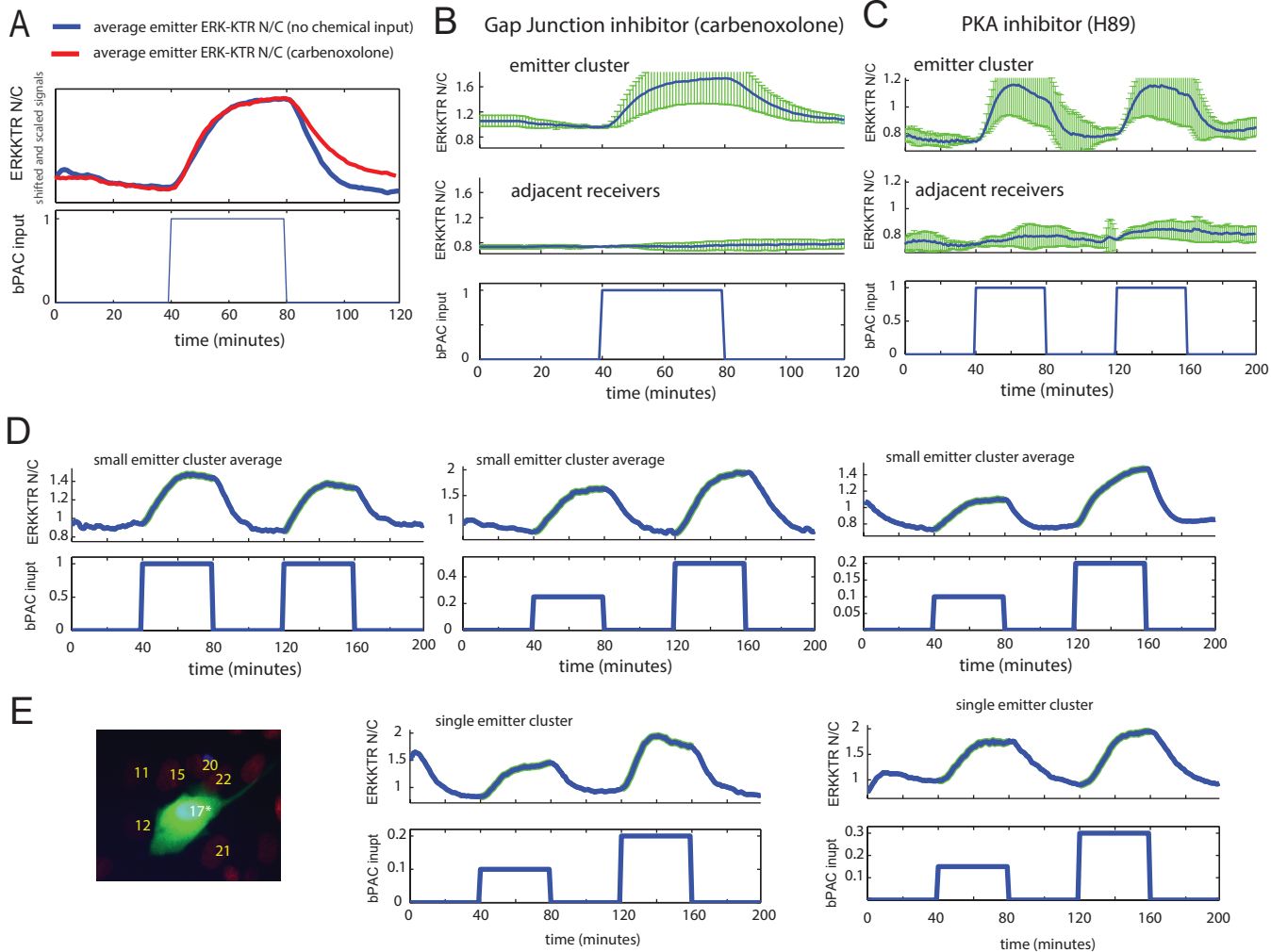

Supplement: S4 Fig — (A) Comparison of ERK-KTR N/C signal decay after bPAC input pulse shutoff. In red (top plot) is the average emitter ERK-KTR N/C signal for the small-emitter-cluster experiment with gap-junction inhibition (carbenoxolone) from Fig 4A. Plotted for comparison (blue) is the average emitter ERK-KTR N/C signal for an all-emitter experiment (no chemical input). Signals are shifted and scaled to have same peak amplitudes in order to be able to directly compare signal decay after the bPAC input pulse (bottom plot) turns off. (B-C) Plots of mean and standard deviation for small-emitter-cluster experiments under different chemical inhibitions. For standard deviation calculations, each single cell ERK-KTR N/C signal is shifted such that the signal at the beginning of the first pulse is zero to remove baseline variability. Standard deviation is calculated to just before the start of the second pulse. This process is the then repeated for the second pulse enforcing the standard deviation is zero at the beginning of each pulse. This is done for both the emitter groups and the receiver groups. Top plot: emitters, Middle plot: adjacent receivers, Bottom plot: bPAC input. (B) Gap junction inhibition (carbenoxolone) experiment from Fig 4A. (C) PKA inhibition (H89) experiment from Fig 4B. (D-E) Experimental results for small and single emitter clusters surrounded by receivers that do not form gap junctions (MDCKII cells). (D) Average emitter ERK-KTR N/C signal for different small emitter clusters each with different bPAC input pulse sequences. (E) Emitter ERK-KTR N/C signal for different single emitter clusters each with different bPAC input pulse sequences. Image depicts the single emitter cluster (receivers do not have ERK-KTR but have a nuclear marker (red nuclei, yellow label)) whose ERK-KTR N/C signal is plotted in the middle panel. (PDF) [file pcbi.1009873.s004.pdf]

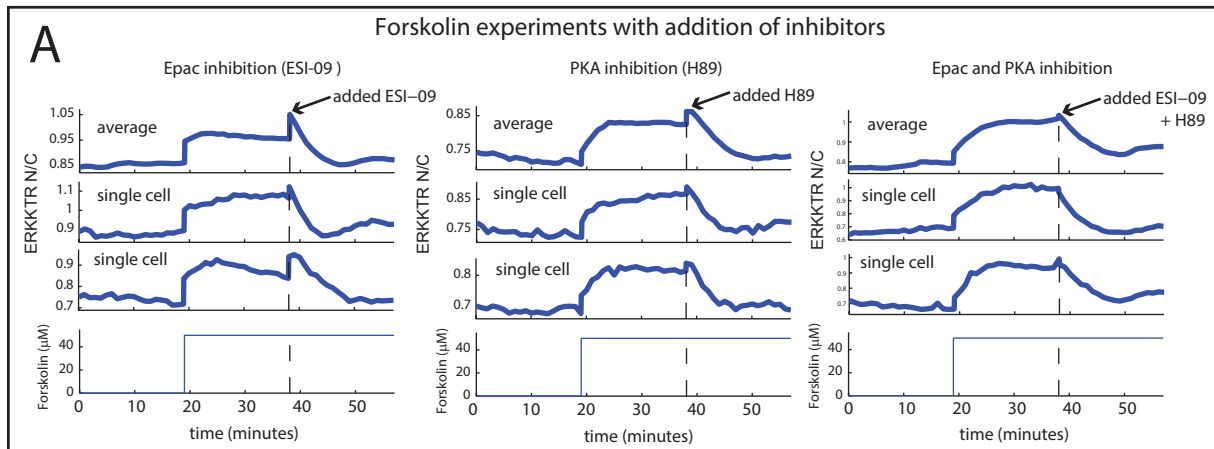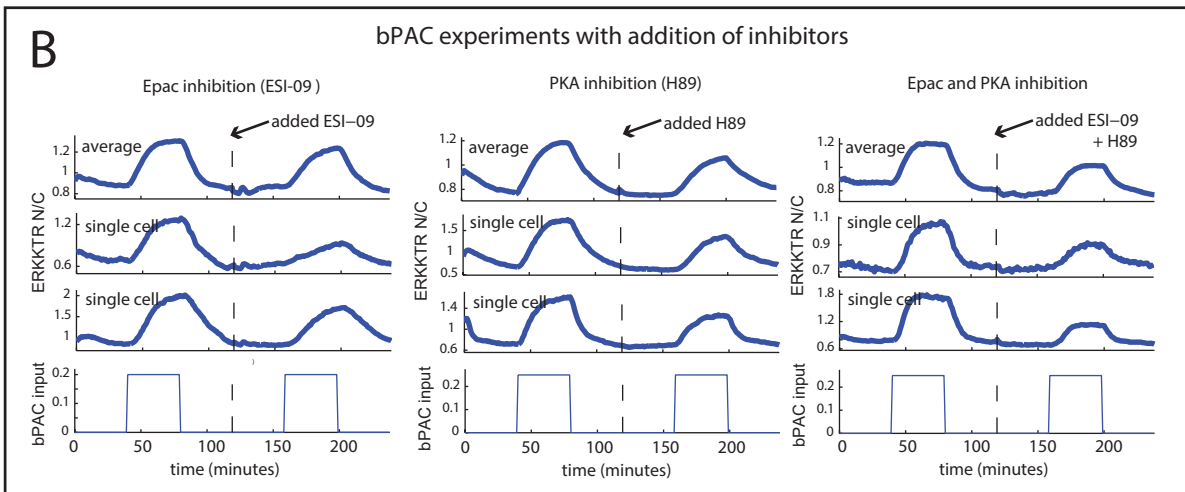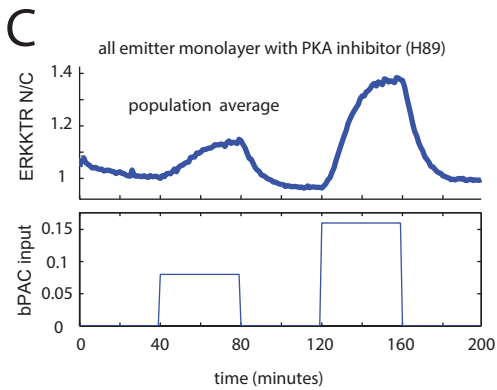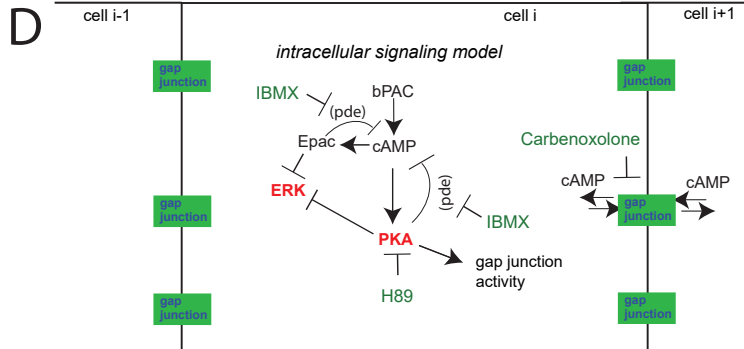

Supplement: S5 Fig — (A) Testing how Epac and PKA inhibitors affect ERK under endogeneous adenalyl cyclase activation through Forskolin (all-receiver experiment). Cells are imaged for twenty mintutes. Forskolin is then added, and the same cells are imaged for twenty minutes. An inhibitor(s) is then added and the sames cells are imaged for the final twenty minutes. All panels: top plot: average ERK-KTR N/C signal, middle plots: single cell ERK-KTR N/C signals, bottom plot: step input of Forskolin. Left Panel: Epac inhbitor (ESI-09), Middle Panel: PKA inibition (H89), Right Panel: both inhibitors (ESI-09 and H89). Note that the addition of Forksolin disturbs the microscope where it can take a few minutes to find and realign the same cells, causing a discontinuity in the measured signal. The same holds true for the addition of the inhibitor(s). (B) Testing how Epac and PKA inhibitors affect ERK under bPAC activation (all-emitter experiment). Cells are imaged during a pulsed bPAC input sequence (40 minutes off, 40 minutes on, 40 minutes off). An inhibitor(s) is than added and the bPAC input sequence sequence is repeated while the same cells are imaged. All panels: top plot: average ERK-KTR N/C signal, middle plots: single cell ERK-KTR N/C signals, bottom plot: bPAC input pulse sequence. Left Panel: Epac inhbitor (ESI-09), Middle Panel: PKA inibition (H89), Right Panel: both inhibitors (ESI-09 and H89). Note that the addition of the inhibitor(s) disturbs the microscope where it can take a few minutes to find and realign the same cells, causing a discontinuity in the measured signal. (C) All-emitter monolayer experiment with PKA inhibition (H89): top plot: average ERK-KTR N/C signal of emitters. bottom plot: bPAC input pulse sequence of increasing amplitudes. The average ERK-KTR N/C signal exhibits similar qualitative behavior to the all-emitter experiment (no PKA inhibition) from Fig 2F. (D) Literature-based illustration of multi-cellular model where each cell has an intracellular circuit an [file pcbi.1009873.s005.pdf]

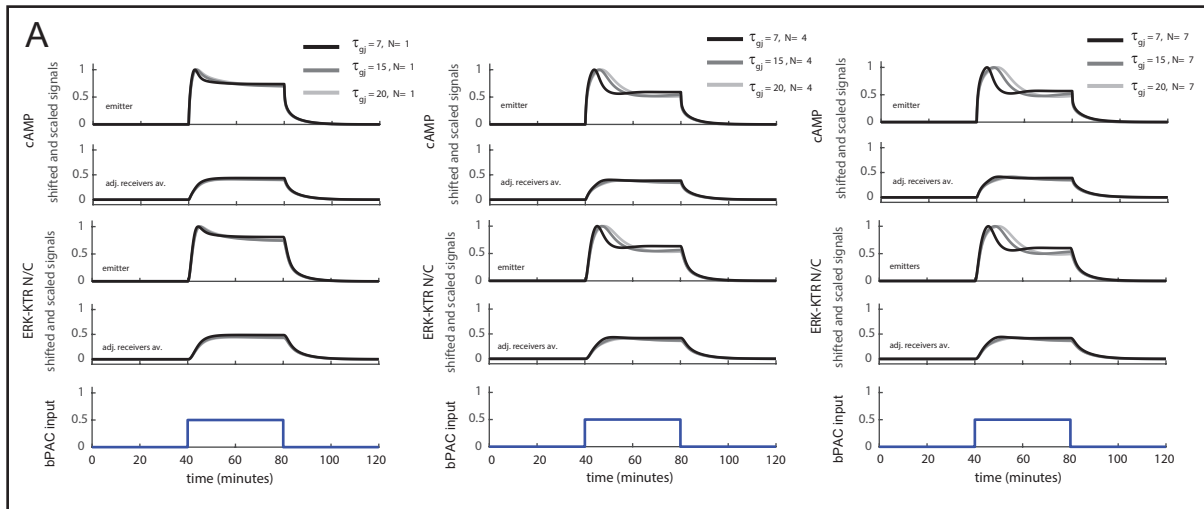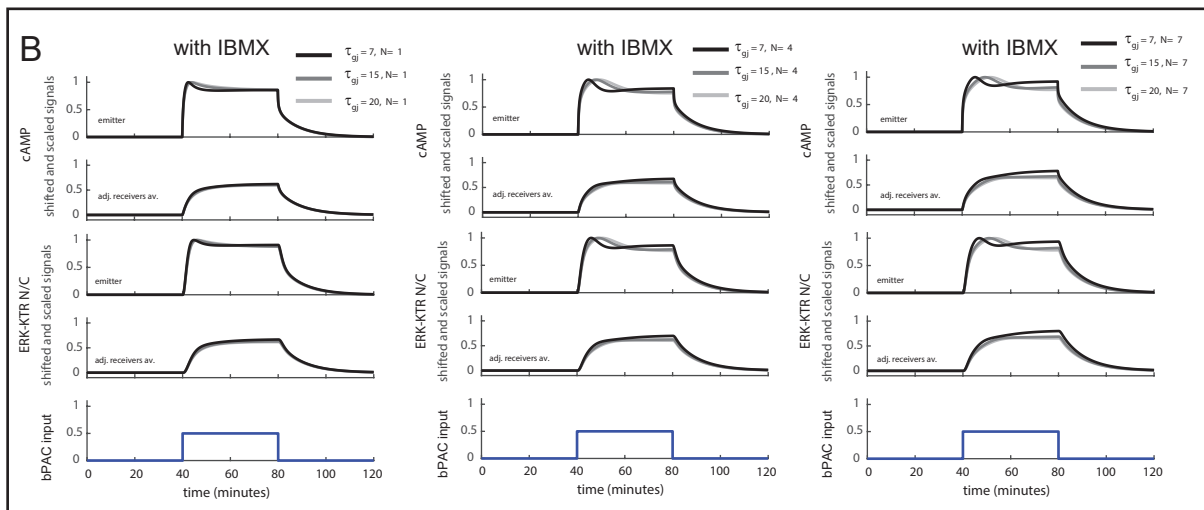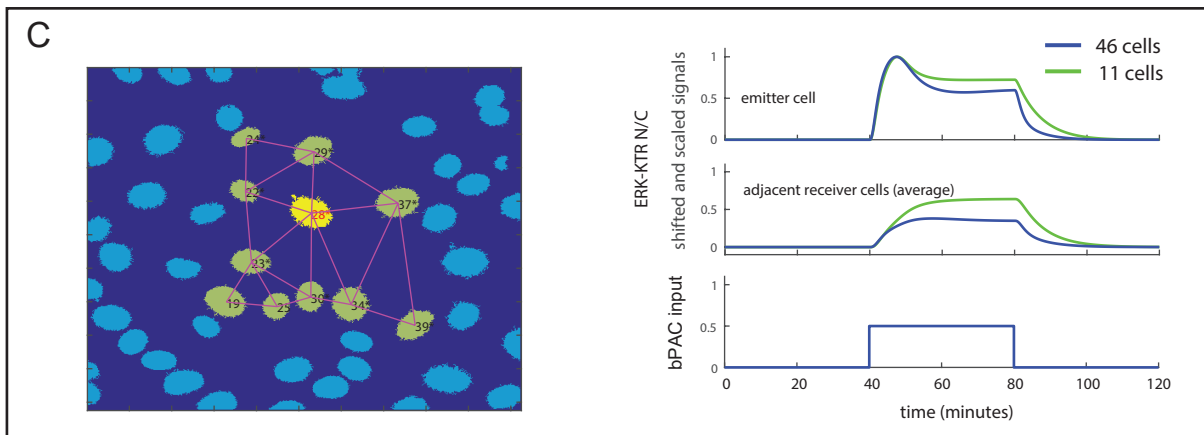

Supplement: S7 Fig — (A-B) Parameter sweep of τgj = 7, 15, 20 and N = 1, 4, 7, where τgj = 15, N = 4 represents the values used for the results in Fig 5I. ERK-KTR N/C and cAMP signals are shifted to zero and scaled for comparison, with the peak of the emitter signal at 1 and the minimum at zero. The receiver signals are multiplied by the same scaling factor as the emitter to maintain the same relative amplitudes. (A) without IBMX. (B) with IBMX. (C) Comparison of model results presented in Fig 5I, which simulates 46 cells, to simulation results that use 11 cells represented in left panel (emitter (yellow nucleus, red label) and receiver cells (light green nuclei, black label), and where lines connecting nuclei represent direct cell-cell communication through gap-junctions). (PDF) [file pcbi.1009873.s007.pdf]

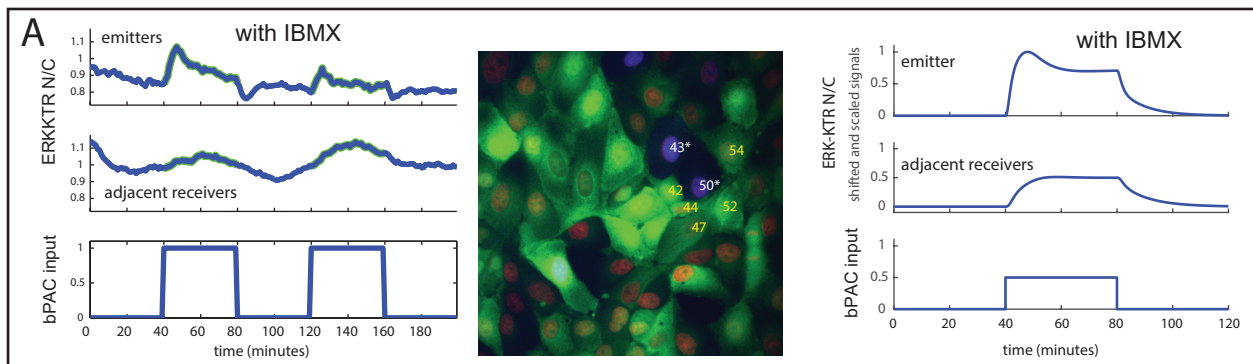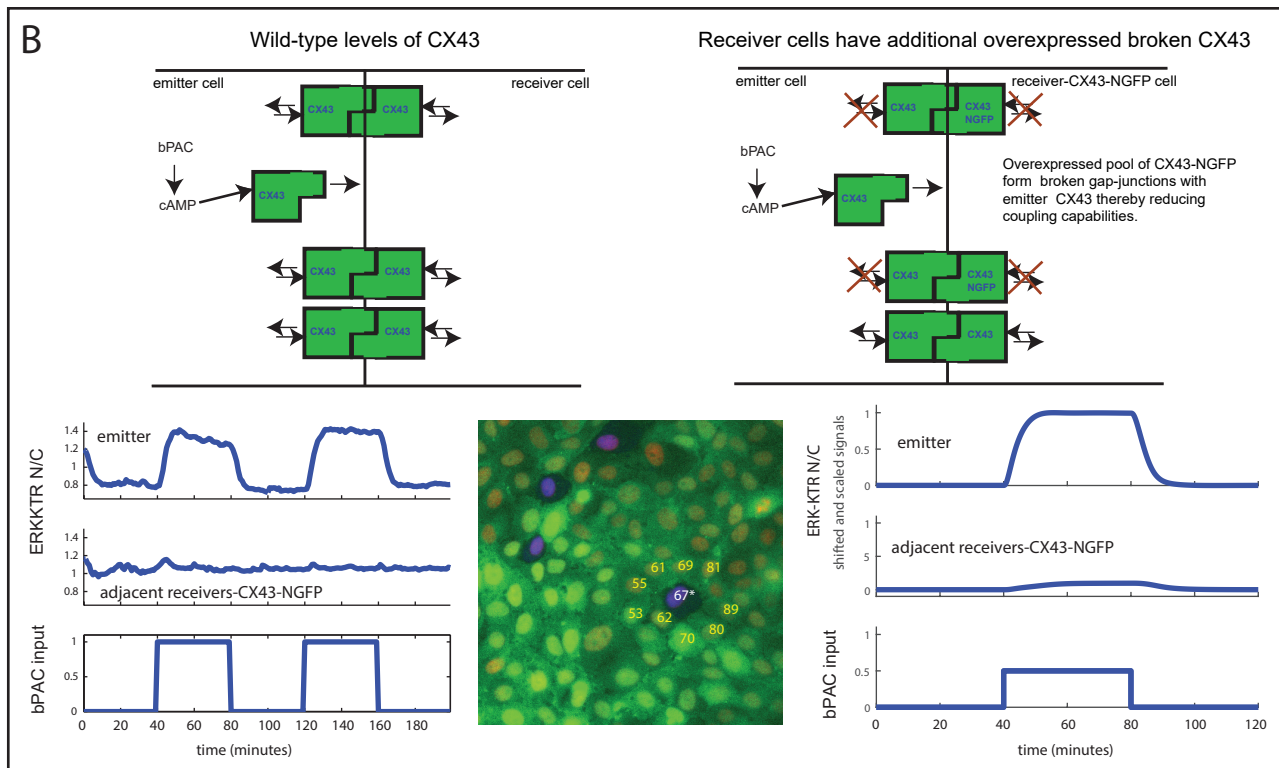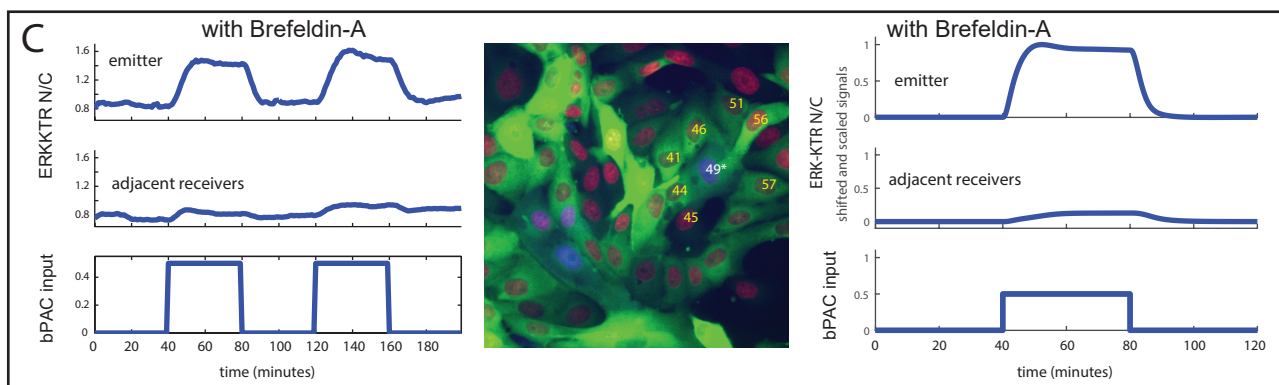

Supplement: S8 Fig — For (A), (C) and bottom 3 panels in (B): Left Panel: top plot: average small-emitter-cluster ERK-KTR N/C signal, middle plot: average adjacent receivers ERK-KTR N/C signal, bottom plot: bPAC input pulse sequence. Middle Panel: image of small emitter cluster (cluster emitters: white labels, adjacent receivers: yellow labels). Right Panel: predicted model results. (A) Experimental observations and model predictions where an emitter in a small emitter cluster exhibits ERK-KTR N/C overshoot under PDE inhibition (IBMX). Same model used from Fig 5I, but with the PDE dependent cAMP decay rate set to zero. (B) Cell-cell coupling models for emitters coupled to different receiver strains. Top left panel: emitter and receiver strains contain the endogenous connexin43. Top right panel: emitter strain coupled to the receiver-CX43-NGFP strain which contains both the endogenous connexin43 and the overexpressed connexin43-NGFP that can form gap-junctions but no flux. Bottom left panel: ERK-KTR N/C signals for small-emitter-cluster and receiver-CX43-NGFP experiment. Over expression of broken connexin43 (CX43-NGFP) reduces cAMP coupling through competition with endogenous connexin43. Bottom right panel: same model used from Fig 5I, but with the max gap junctional transport rate of cAMP set to .2 that of the wild-type to capture the reduced coupling effect. (C) Emitter in single emitter cluster shows large amplitude with very little overshoot in the ERK-KTR N/C signal in the presence of the trafficking inhibitor Brefeldin-A. A small amount of coupling to receiver cells is observed. Same model used from Fig 5I, but with the max gap junctional transport rate of cAMP set to .25 that of the wild-type to capture the reduced coupling effect. (PDF) [file pcbi.1009873.s008.pdf]

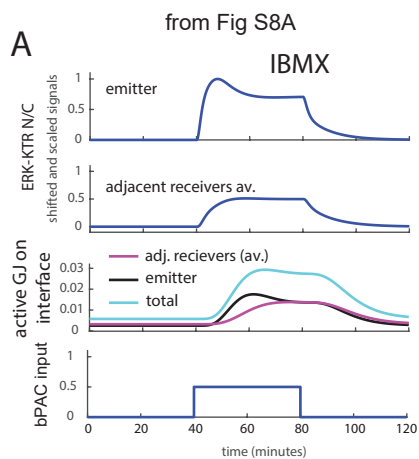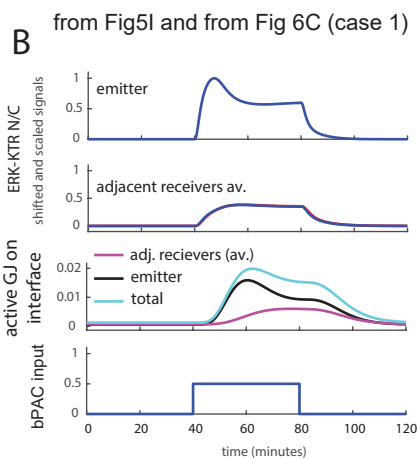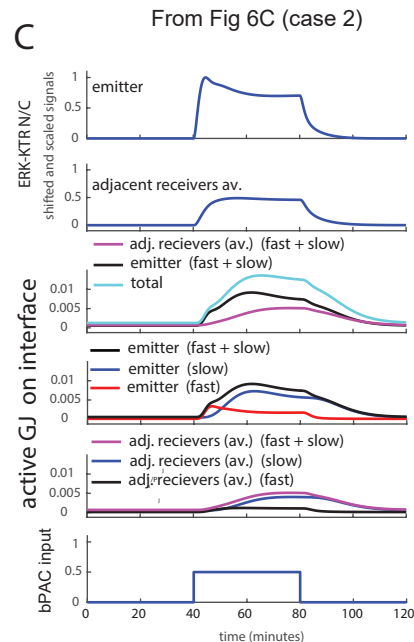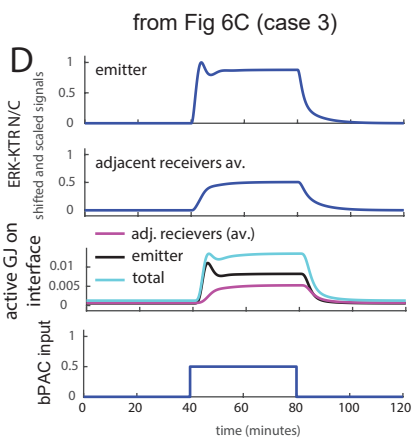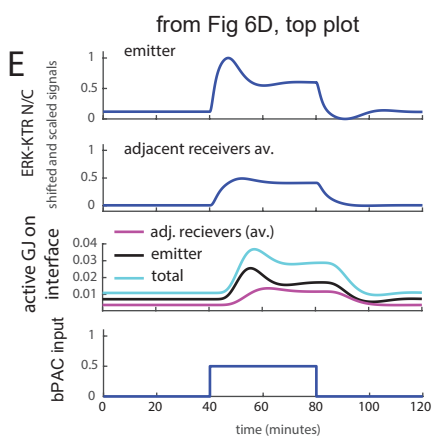

Supplement: S9 Fig — (A)-(E) Active gap-junction state dynamics at the emitter/receivers interface for the modeled single-emitter-cluster for various cases throughout the paper. Top plot: emitter ERK-KTR N/C signal (shifted and scaled). Second plot from the top: average of adjacent receivers ERK-KTR N/C signal (shifted and scaled by same factor as emitter). Third plot from the top: corresponding cAMP/PKA driven dynamics of the active gap junctions on the emitter/receiver interfaces (averaged). Also included is a breakdown of the contributions from the emitter and the average of the adjacent receivers. Bottom plot: bPAC input pulse sequence. (A) Model presented in S8(A) Fig. Same model as Fig 5I (τgj = 15, N = 4), but in the presence of PDE inibitor IBMX. (B) Model presented in Fig 5I (τgj = 15, N = 4) as well as in Fig 6C (case 1, slow population only). (C) Model presented in Fig 6C (case 2, slow and fast population mixture). Fourth plot from the top: a breakdown of the contribution from the emitter into its slow and fast populations. Fifth plot from the top: a breakdown of the contribution from the average of the adjacent receivers into its slow and fast populations. (D) Model presented in Fig 6C (case 3, fast population only). (E) Model which accounts for undershoot of the ERK-KTR N/C signal corresponding to Fig 6D (top plot, no IBMX, no PKA regulation of AC). (PDF) [file pcbi.1009873.s009.pdf]

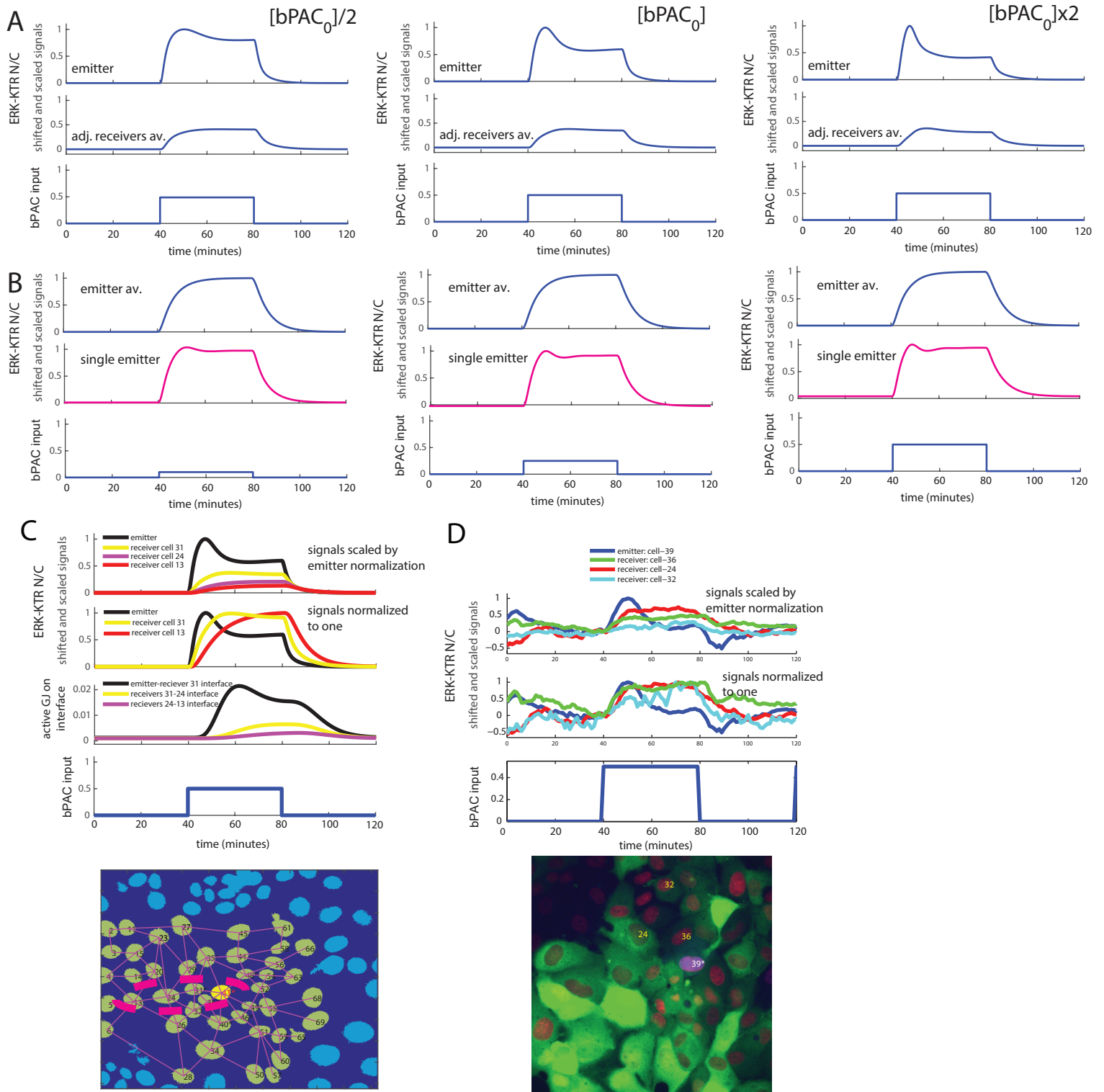

Supplement: S10 Fig — (A) Varying the bPAC expression level for the emitter in the single-emitter-cluster model where [bPAC0] has been the implicit concentration throughout the paper. And where the value of βc,b (cAMP production rate due to bPAC) is proportional to the bPAC expression level. Presented are results for expression levels [bPAC0]/2, [bPAC0], and [bPAC0] × 2. Note that this is effectively the same as scaling a given bPAC input amplitude by the same factor. (B) Simulating cell-cell heterogeneity in bPAC expression across an all-emitter monolayer for different bPAC input pulse amplitudes. Cell-to-cell differences (gradients) in bPAC expression can yield single emitters with small amounts of overshoot (middle plot) relative to the average (top plot). (C)-(D) Top panel: For top plot, ERK-KTR N/C signals are shifted to zero at the beginning of the bPAC input pulse and scaled by the resulting peak emitter amplitude which results in the peak of the emitter signal at 1. The receiver signals, multiplied by the same scaling factor as the emitter, maintain the same relative amplitudes (proportionally). Second plot from top: same as top plot but all plotted signals have their peak normalized to 1 to better examine delays. Bottom plot: bPAC input. Bottom panel: Image of multicellular system from which signals are measured. (C) Model example of ERK-KTR N/C signal propagation from a single-emitter-cluster. Third plot from top: corresponding dynamics of the active gap junctions on the cell-cell interfaces for the cell-cell pairs measured. In bottom panel: emitter (yellow nucleus, red label), receivers (light green nuclei, black label). (D) Experimental example of ERK-KTR N/C signal propagation from a single-emitter-cluster experiment. In bottom panel: emitter (white label), receivers (yellow label). (PDF) [file pcbi.1009873.s010.pdf]

A

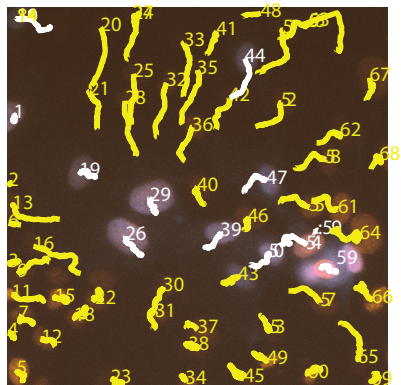

B

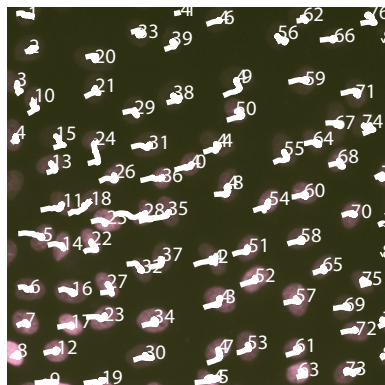

C

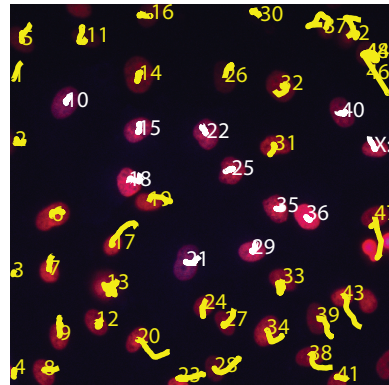

D

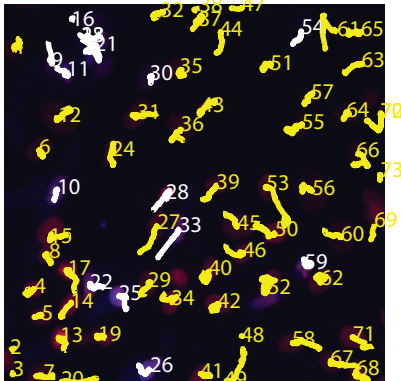

E

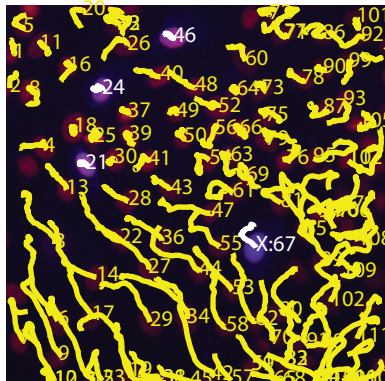

F

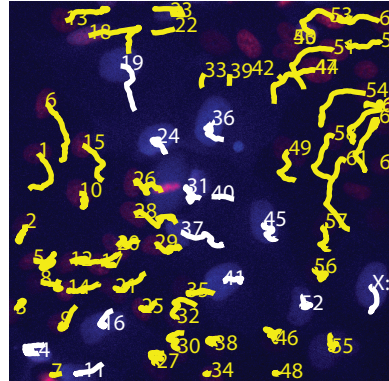

Supplement: S11 Fig — And the phenotype is potentially regulated through PKA. (A-F) Illustration depicts overall mobility of cells over the course of an experiment. The image captures the final positions of the nuclei (emitters (white label), receivers (yellow label)) at the end of the experiment overlayed with their individual mobility trajectories that occurred over the duration of the experiment. (A) Small-emitter-cluster experiment (Fig 3A) exhibiting emitter/receiver cAMP coupling over a 2 hour and 40 minute duration. (B) All-emitter experiment (Fig 2B) over a 3 hour and 20 minute duration. (C) Gap-junction inhibition experiment (carbenoxolone, Fig 4A) exhibiting no emitter/receiver cAMP coupling over a 2 hour duration. (D) PKA inhibition experiment (Fig 4B) exhibiting little to no emitter/receiver cAMP coupling over a 3 hour and 20 minute duration. (E) Small emitter cluster and receiver-C43-NGFP experiment (S8(B) Fig) exhibiting reduced emitter/receiver cAMP coupling over a 3 hour and 20 minute duration. (F) Small emitter-C43-NGFP cluster and receiver experiment exhibiting reduced emitter/receiver cAMP coupling over a 2 hour and 20 minute duration. (PDF) [file pcbi.1009873.s011.pdf]
