# Supplementary material for: Analysis of localized cAMP perturbations within a tissue reveal the effects of a local, dynamic gap junction state on ERK signaling
Source: PLoS Comput Biol. 2022 Mar 30;18(3):e1009873. doi: 10.1371/journal.pcbi.1009873 (PMC9000136; doi:10.1371/journal.pcbi.1009873)
Supplement: S6 Fig — (A-E) Modeling results for simplified intracellular pathway with no feedbacks in an all-emitter monolayer. Model is fitted to ERK-KTR N/C data for 6 minute bPAC input pulses applied to an all-emitter monolayer (S1(B) Fig, IBMX, population average). We present results for two values of the deactivation rate of ERK γerk using the expression κerkγerk where κerk is a scaling factor and γerk is a fitted deactivation rate. ERK-KTR N/C and cAMP signals are shifted to zero and scaled for comparison, with the peak of the emitter signal at 1 and minimum at zero. (A) ERK-KTR N/C signal and predicted cAMP signal driven by 6 minute bPAC input pulse. Upper plot: fitted model (κerk = 1). Middle plot: same fit except for a slower ERK deactivation rate reduced by a factor κerk = .5. (B) Same as (A) but with IBMX. (C) Same plots as (B) but with the ERK-KTR N/C data overlayed to show the fit for both the κerk = 1 and κerk = .5 cases. (D) Same as (A) but for 40 minute bPAC input pulses. (E) Same as (B) but for 40 minute bPAC input pulses with IBMX. (F) PKA signal for 40 minute bPAC input pulses (from same modeling results as (D)). (G-H) ERK-KTR N/C and cAMP signals are shifted to zero and scaled for comparison, with the peak of the emitter signal at 1 and the minimum at zero. The receiver signals are multiplied by the same scaling factor as the emitter to maintain the same relative amplitudes. (G) Single-emitter-cluster modeling results for the model presented in Fig 5G, but where the gap junctional permeability is reduced by a factor of 3 (κgj = 1/3) or increased by a factor of 3 (κgj = 3). (H) Single-emitter-cluster modeling results for the model presented in Fig 5H, but where the gap junctional permeability is reduced by a factor of 3 (κgj = 1/3) or increased by a factor of 3 (κgj = 3). (I) All-emitter modeling results for the intracellular circuit with PKA feedback through the PDEs presented in Fig 5H. In top plot, the ERK-KTR N/C and cAMP signals are shifted to zero and scaled for [file pcbi.1009873.s006.pdf]

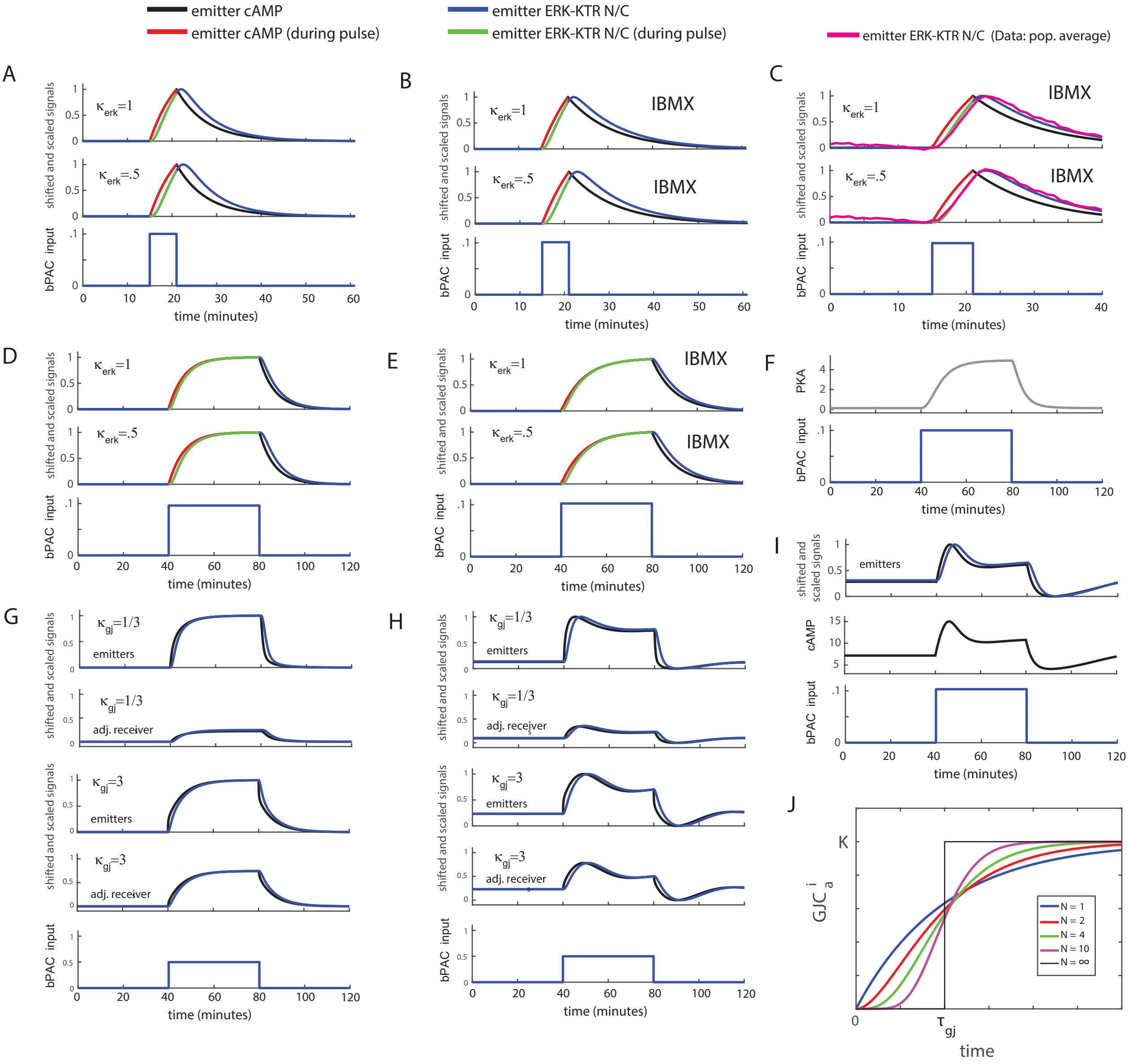
emitter cAMP
emitter cAMP (during pulse)
emitter ERK-KTR N/C
emitter ERK-KTR N/C (during pulse)
emitter ERK-KTR N/C (Data: pop. average)
A
B
C
D
E
F
G
H
I
J
κerk=1
κerk=.5
IBMX
κgj=1/3
κgj=3
emitters
adj. receiver
shifted and scaled signals
bPAC input
PKA
cAMP
time (minutes)
GJC
K
τgj
time
N = 1
N = 2
N = 4
N = 10
N = ∞
