# Supplementary material for: Analysis of localized cAMP perturbations within a tissue reveal the effects of a local, dynamic gap junction state on ERK signaling
Source: PLoS Comput Biol. 2022 Mar 30;18(3):e1009873. doi: 10.1371/journal.pcbi.1009873 (PMC9000136; doi:10.1371/journal.pcbi.1009873)
Supplement: S1 Table — Table of observations associated with the mobility phenotype under different pharmacological and genetic (emitter or receiver) perturbations. The table is referenced in the Discussion section with deeper discussion in S5 Text. (PDF) [file pcbi.1009873.s017.pdf]

S1 Table. **The effect of chemical and genetic perturbations on the mobility phenotype**

**cell lines**	**chemical perturbation**	**cell-cell coupling**	**observed mobility phenotype?, experimental frequency**
all emitters	none	yes	No, 0/17 experiments:<br>12 with no mobility and a search-induced ERK N/C signal at t=0,<br>5 with no mobility and no search-induced ERK N/C signal at t=0
all emitters	PKA inhibition	no	Yes, 1/6 experiments:<br>1 with early mobility start and no search-induced ERK N/C signal at t=0,<br>1 with no mobility and a search-induced ERK N/C signal at t=0,<br>4 with no mobility and no search-induced ERK N/C signal at t=0
all receivers	none	yes	No, 0/4 experiments:<br>4 with no mobility and no search-induced ERK N/C signal at t=0
small emitter cluster/receivers	none	yes	Yes, 8/13 experiments:<br>5 with early mobility start and a search-induced ERK N/C signal at t=0,<br>3 with mid-to-late mobility start and a search-induced ERK N/C signal at t=0,<br>3 with no mobility and a search-induced ERK N/C signal at t=0,<br>2 with no mobility and no search-induced ERK N/C signal at t=0
single emitter cluster/receivers	none	yes	Yes, 4/20 experiments:<br>2 with early mobility start and a search-induced ERK N/C signal at t=0,<br>1 with first-pulse mobility start and a search-induced ERK N/C signal at t=0,<br>1 with first-pulse mobility start and no search-induced ERK N/C signal at t=0,<br>8 with no mobility and a search-induced ERK N/C signal at t=0,<br>8 with no mobility and no search-induced ERK N/C signal at t=0
small emitter cluster/receivers	gap-junction inhibition	no	No, 0/2 experiments:<br>2 with no mobility and no search-induced ERK N/C signal at t=0
small emitter cluster/receivers	PKA inhibition	reduced, low flux	Yes, 0/6:<br>1 with no mobility and a search-induced ERK N/C signal at t=0,<br>5 with no mobility and no search-induced ERK N/C signal at t=0
small emitter cluster/ receivers-CX43-NGFP	none	reduced, low flux	Yes, 7:13<br>2 with early mobility start and a search-induced ERK N/C signal at t=0,<br>4 with mid-to-late mobility start and a search-induced ERK N/C signal at t=0,<br>1 with first pulse mobility start and no search-induced ERK N/C signal at t=0,<br>4 with no mobility and a search-induced ERK N/C signal at t=0,<br>2 with no mobility and no search-induced ERK N/C signal at t=0
small emitters-CX43-NGFP cluster/receivers	none	reduced, low flux	Yes, 1:1<br>1 with first pulse mobility start and a search-induced ERK N/C signal at t=0
small emitter cluster/ receivers-MDCKII (no gap junc.)	none	no	No, 2:17<br>2 with early mobility start and a search-induced ERK N/C signal at t=0,<br>9 with no mobility and a search-induced ERK N/C signal at t=0,<br>6 with no mobility and no search-induced ERK N/C signal at t=0,
