# Supplementary material for: Analysis of localized cAMP perturbations within a tissue reveal the effects of a local, dynamic gap junction state on ERK signaling
Source: PLoS Comput Biol. 2022 Mar 30;18(3):e1009873. doi: 10.1371/journal.pcbi.1009873 (PMC9000136; doi:10.1371/journal.pcbi.1009873)
Supplement: S2 Table — Table of parameters used for the multicellular simulation results presented in Fig 5. (PDF) [file pcbi.1009873.s018.pdf]

S2 Table. **Simulation parameter values used for results in Fig 5**

**Fig./ Mod.**	c_b^i	$\beta_{c,b}$	$\beta_{c,ac}$	X_{f_1}	$c_{ac,pk}$	γ_c	γ_{c,pd_b}	$\gamma_{c,pd2}$	X_{f_2}	k_{gj}	$k_{gj,gjf}$
5B/M	1 (E)	0.3788	0.0095	0.5000	0	0.0017	8.3333×10^{-4}	–	–	–	–
5C -E/M	1 (E)	0.3788	0.0095	0.5000	0	0.0017	$S_{\gamma_{c,pd_b}}$	–	–	–	–
5G/M	0 (R) 1 (E)	0.3788	0.0095	0.5000	0	0.0017	8.3333×10^{-4}	–	–	0.0312	–
5H/IO	0 (R) 1 (E)	0.4167	0.0208	–	0	0.0010	–	0.0075	0.0050	0.0625	–
5I/M	0 (R) 1 (E)	0.3788	0.0095	0.5000	0	0.0017	8.3333×10^{-4}	–	–	0.0104	0.2083

**Fig./ Mod.**	X_{f_3}	$k_{pk,c}$	X_{f_5}	n_{f_5}	γ_{pk}	$\gamma_{pk,pd}$	X_{f_4}	$k_{pd,pk}$	γ_{pd}	k_e	γ_e
5B/M	–	4.5753×10^{-8}	1000	4	0.0125	–	–	–	–	S_{k_e}	0.0042
5C-E/M	–	4.5753×10^{-8}	1000	4	0.0125	–	–	–	–	0.0292	0.0042
5G/M	–	4.5753×10^{-8}	1000	4	0.0125	–	–	–	–	0.0292	0.0042
5H/IO	–	1.3021×10^{-10}	4200	4	0.0042	0.0313	7.5	0.0021	0.0010	0.0146	0.0021
5I/M	1	4.5753×10^{-8}	1000	4	0.0125	–	–	–	–	0.0292	0.0042

**Fig./ Mod.**	$\gamma_{e,c}$	X_{f_6}	γ_{ek}	$k_{ek,e}$	$\beta_{gj,pk}$	X_{f_7}	N	τ_{gj}	γ_{gj}		
5B/M	0.0167	100	0.0167	2.0833×10^{-4}	–	–	–	–	–		
5C-E/M	0.0167	100	0.0167	2.0833×10^{-4}	–	–	–	–	–		
5G/M	0.0167	100	0.0167	2.0833×10^{-4}	–	–	–	–	–		
5H/IO	0.0042	100	0.0167	2.0833×10^{-4}	–	–	–	–	–		
5I/M	0.0167	100	0.0167	2.0833×10^{-4}	0.0208	10	4	15	0.0521		

M corresponds to the Minimal model. *IO* corresponds to Intracellular Overshoot model.
$S_{k_e} = 0.0292$ or 0.0146.
$S_{\gamma_{c,pd_b}} = 8.3333 \times 10^{-4}$ (no IBMX) or 0 (IBMX).
