# Supplementary material for: Analysis of localized cAMP perturbations within a tissue reveal the effects of a local, dynamic gap junction state on ERK signaling
Source: PLoS Comput Biol. 2022 Mar 30;18(3):e1009873. doi: 10.1371/journal.pcbi.1009873 (PMC9000136; doi:10.1371/journal.pcbi.1009873)
Supplement: S3 Table — Table of parameters used for the multicellular simulation results presented in S8 Fig and Fig 6. (PDF) [file pcbi.1009873.s019.pdf]

S3 Table. **Simulation parameter values used for results in S9 Fig and Fig 6**

	c_b^i	$\beta_{c,b}$	$\beta_{c,ac}$	X_{f_1}	$c_{ac,pk}$	γ_c	γ_{c,pd_b}	$\gamma_{c,pd2}$	X_{f_2}	k_{gj}	$k_{gj,gjf}$
**Fig./ Mod.**											
S9A/M	0 (R) 1 (E)	0.3788	0.0095	0.5000	0	0.0017	0	–	–	0.0104	0.2083
S9B/M	0 (R) 1 (E)	0.3788	0.0095	0.5000	0	0.0017	8.3333 $\times 10^{-4}$	–	–	0.0104 $\times .2$	0.2083 $\times .2$
S9C/M	0 (R) 1 (E)	0.3788	0.0095	0.5000	0	0.0017	8.3333 $\times 10^{-4}$	–	–	0.0104 $\times .25$	0.2083 $\times .25$
S9D/M	0 (R) 1 (E)	0.3788	0.0095	0.5000	0	0.0017	8.3333 $\times 10^{-4}$	–	–	0.0104 $\times .25$	0.2083 $\times .25$
6C/M	0 (R) 1 (E)	0.3788	0.0095	0.5000	0 or 1	0.0017	8.3333 $\times 10^{-4}$	–	–	0.0104	0.2083

	X_{f_3}	$k_{pk,c}$	X_{f_5}	n_{f_5}	γ_{pk}	$\gamma_{pk,pd}$	X_{f_4}	$k_{pd,pk}$	γ_{pd}	k_e	γ_e
**Fig./ Mod.**											
S9A/M	1	4.5753 $\times 10^{-8}$	1000	4	0.0125	–	–	–	–	0.0292	0.0042
S9B/M	1	4.5753 $\times 10^{-8}$	1000	4	0.0125	–	–	–	–	0.0292	0.0042
S9C/M	1	4.5753 $\times 10^{-8}$	1000	4	0.0125	–	–	–	–	0.0292	0.0042
S9D/M	1	4.5753 $\times 10^{-8}$	1000	4	0.0125	–	–	–	–	0.0292	0.0042
6C/M	1	4.5753 $\times 10^{-8}$	1000	4	0.0125	–	–	–	–	0.0292	0.0042

	$\gamma_{e,c}$	X_{f_6}	γ_{ek}	$k_{ek,e}$	$\beta_{gj,pk}$	X_{f_7}	N	τ_{gj}	γ_{gj}		
**Fig./ Mod.**											
S9A/M	0.0167	100	0.0167	2.0833 $\times 10^{-4}$	0.0208	10	4	15	0.0521		
S9B/M	0.0167	100	0.0167	2.0833 $\times 10^{-4}$	0.0208	10	4	15	0.0521		
S9C/M	0.0167	100	0.0167	2.0833 $\times 10^{-4}$	0.0208	10	4	1*, 15*	0.0521		
S9D/M	0.0167	100	0.0167	2.0833 $\times 10^{-4}$	0.0208	10	4	1	0.0521		
6C/M	0.0167	100	0.0167	2.0833 $\times 10^{-4}$	0.0208	10	4	1*, 15*	0.0521		

M corresponds to the Minimal model.
For S9C Fig and Fig 6C, the 1^*, 15^* under τ_{gj} refers to the case of the mixture of fast (1 minute delay) and slow (15 minute delay) gap junction populations whose modeling results are presented in S9C Fig and Fig 6C (case 2).
