# Supplementary material for: Analysis of localized cAMP perturbations within a tissue reveal the effects of a local, dynamic gap junction state on ERK signaling
Source: PLoS Comput Biol. 2022 Mar 30;18(3):e1009873. doi: 10.1371/journal.pcbi.1009873 (PMC9000136; doi:10.1371/journal.pcbi.1009873)
Supplement: S1 Text — This section further explores the operational regimes of bPAC and the ERK-KTR reporter that were presented in Fig 2 and S1 Fig. It also further discusses the choice of the ERK-KTR reporter as a downstream reporter of cAMP/PKA activity. (PDF) [file pcbi.1009873.s020.pdf]

S1 Text. Exploring the operational regimes of bPAC and the ERK-KTR reporter

To build upon the discussion in the main text (from section 'The combination of bPAC and the ERK-KTR reporter provides quantitative single cell ERK activity measurements that are highly sensitive to intracellular cAMP/PKA dynamics'), we further explored the behavior and the operational regime of the bPAC/cAMP/ERK-KTR pathway for all-emitter monolayer experiments.

As a means of further testing the ERK-KTR N/C signal's sensitivity to bPAC/cAMP dynamics, we applied a sequence of short bPAC input pulses (6 minute pulse width with 30 minutes off between pulses, and increasing pulse amplitudes) to an all-emitter monolayer. We observed that within a minute after each bPAC input pulse turned on, the ERK-KTR N/C signal rose during the pulse in the single cell ERK-KTR N/C signals (S1B Fig, single cell plots: green segment indicates when pulse is on). This mirrors the initial linear accumulation of cAMP that is driven by bPAC, whose activity reaches a constant level within seconds of the bPAC input pulse (blue light) turning on [1], and before degradation becomes a significant loss term relative to the bPAC production rate of cAMP. When the bPAC input pulse (blue light) shuts off, where bPAC molecules can take an additional 30 seconds to deactivate [1], we typically observe the ERK-KTR N/C signal to start decaying a minute or so after that. And as expected, the IBMX case decays slower than the wild-type case (S1B Fig, top plot, compare average signals).

While the expected cAMP time dynamics are captured, we are also interested in how linear the response is to bPAC input amplitude as well as where it saturates. Thus, for only these experiments (S1B Fig) the neutral density filter was removed in order to maximize blue light photon flux whose magnitude is $\alpha F_{\max}$. Here the maximum blue light flux is $F_{max} \approx 200\ \mu\text{W cm}^{-2}$ in the presence of the neutral density filter, but is scaled by α when there is no neutral density filter and where $\alpha \approx 7$ (see Materials and Methods for details). To visualize the linear and saturated response regions, we define the metric ΔERK-KTR N/C response as the difference between the ERK-KTR N/C signal at the start of the pulse and its value at a specific time during the pulse, typically set as the time of the end of the pulse (see visual example in S1C Fig, second plot from the top). For the sequence of short bPAC input pulses of ascending amplitude $.2\alpha F_{\max}$, $.4\alpha F_{\max}$, $.6\alpha F_{\max}$ and $\alpha F_{\max}$, we observe variability across single cells in the linear range of ΔERK-KTR N/C response versus bPAC input amplitude (S1B Fig, right plots). For example, for one cell, its linear range is only up to a peak input amplitude of $.2\alpha F_{\max}$ while for another cell, it is $.6\alpha F_{\max}$. Above a given cell's peak input amplitude defining its linear range, one observes saturation. There are different potential sources of saturation: 1) bPAC activity saturates above some level of blue light intensity, and 2) one of the components of the cAMP/ePAC/ERK-KTR pathway significantly saturates above some level of cAMP. Importantly, we plotted the ΔERK-KTR N/C signal versus bPAC activity amplitude at 2 minutes into the pulse and there is still saturation above the same peak amplitude for a given cell. This indicates that bPAC activity is saturated for these higher amplitudes and not the ERK-KTR reporter. This saturation effect also manifests in the same initial rate of rise of the ERK-KTR N/C signal across the pulses where the bPAC activity is saturated. Thus, identical initial rates of rise of the ERK-KTR N/C signal across different input amplitude is a signature of bPAC saturation. And as seen in the single cell data, the bPAC input amplitude for when this occurs varies from cell to cell.

An experimental example where bPAC does not get saturated for a bPAC input sequences of increasing amplitudes are illustrated in S1C Fig. Here one can see the initial rate of rise of the ERK-KTR N/C signal is proportional to the bPAC input amplitude across many of the single cells presented. The steady state ERK-KTR N/C signal at 40 minutes is proportional for many as well (S1C Fig, ΔERK-KTR N/C versus bPAC input amplitude, right plots). However, we do find cases of ERK-KTR N/C amplitude saturation. S1F Fig shows ERK-KTR N/C signal for a bPAC input pulse sequence with increasing amplitudes. While the initial rate of rise of the ERK-KTR signal is proportional for the two pulses, the steady state amplitude reached is not, suggesting saturation especially for the response in the second pulse. We want to emphasize the importance in measuring the rise ERK-KTR N/C signal as a function of the bPAC input pulse amplitude as the first measurement of proportionality. The second measurement of proportionality is if the steady state amplitude reached during the pulse is proportional to the bPAC input pulse amplitude. If both are proportional then the system is operating within its dynamic range for that given cell. Furthermore, in S1F Fig the ERK-KTR N/C signal shows little to no delay in shutting off at a maximum rate after the first, smaller amplitude pulse shuts off. For the second, higher amplitude pulse, there is a notable delay of a couple extra minutes in the ERK-KTR N/C signal until it reaches shutoff at a maximum rate. Delay after shutoff of a few minutes or more indicates saturation effects in

the ERK-KTR signal.

For all-emitter monolayers experiencing peak bPAC activity (max bPAC input amplitude) with the addition of the PDE inhibitor IBMX that increases the half life of cAMP, we can push at least a portion of the cells to strong saturation in the ERK-KTR N/C signal, typically occurring above a value of 1.8 (S1E Fig). For this emitter there is a large delay between when the bPAC input shuts off and when the ERK-KTR N/C signal starts decaying. And the slope when it does decay is much smaller than the initial rise. This signifies that until cAMP gets below the saturation level where the ERK-KTR reporter plateaus, the ERK-KTR N/C signal cannot decay.

Given the various signatures of saturation for bPAC and the ERK-KTR N/C signal, we design our experiments so that most of the cells remain within the operational regime of the system. And finally, the ERK-KTR reporter in an all-receiver monolayer does not respond to blue light (S1G Fig). Thus in our blue light experiments, ERK-KTR responds to cAMP produced from blue-light induced bPAC, and not to blue light itself.

Throughout this work we use the ERK-KTR N/C signal as a downstream reporter of cAMP/PKA dynamics. Reporters such as the PRKACA (PKA reporter), an over-expressed PKA catalytic subunit (tagged with GFP), are less ideal due to how their signal is quantified (changes in foci fluorescence) and their necessary over expression which can impact the endogenous cAMP/PKA pathway behavior since they interact directly with cAMP. As a result we mainly use the PRKACA reporter to demonstrate qualitative correlation between bPAC/cAMP and PKA activity. While cAMP positively regulates PKA [2], it also does so for Epac [3]. This has led to the development of intracellular cAMP sensors through FRET-based interactions with Epac [4], And similar to the PRKACA reporter, the Epac-FRET sensor is over expressed. As with PRKACA, the Epac-FRET sensor interacts directly with cAMP and thus can potentially alter endogenous cAMP signaling and effector interactions (Epac, PKA, and gated cyclic-nucleotide gated channels) by acting as a sink of cAMP. On the other hand, the ERK-KTR has robust quantification and dynamic range, and is insulated from direct cAMP and PKA interactions. Given that we are only perturbing cAMP levels, this makes the ERK-KTR a good, sensitive downstream reporter of the cAMP/PKA pathway.

References

1. Stierl M, Stumpf P, Udwari D, Gueta R, Hagedorn R, Losi A, et al. Light modulation of cellular cAMP by a small bacterial photoactivated adenylyl cyclase, bPAC, of the soil bacterium Beggiatoa. J Biol Chem. 2011;286(2):1181-1188.
2. Mizuno R, Kamioka Y, Kabashima K, Imajo M, Sumiyama K, Nakasho E, et al. In vivo imaging reveals PKA regulation of ERK activity during neutrophil recruitment to inflamed intestines. Journal of Experimental Medicine. 2014;211(6):1123-1136. doi:10.1084/jem.20132112.
3. Nedvetsky PI, Kwon SH, Debnath J, Mostov KE. Cyclic AMP regulates formation of mammary epithelial acini in vitro. Mol Biol Cell. 2012;23(15):2973-2981.
4. Klarenbeek J, Goedhart J, van Batenburg A, Groenewald D, Jalink K. Fourth-generation epac-based fret sensors for camp feature exceptional brightness, photostability and dynamic range: Characterization of dedicated sensors for flim, for ratiometry and with high affinity. PLOS ONE, 10(4):1-11, 04 2015.
