# Supplementary material for: Analysis of localized cAMP perturbations within a tissue reveal the effects of a local, dynamic gap junction state on ERK signaling
Source: PLoS Comput Biol. 2022 Mar 30;18(3):e1009873. doi: 10.1371/journal.pcbi.1009873 (PMC9000136; doi:10.1371/journal.pcbi.1009873)
Supplement: S2 Text — This section goes through the statistical analysis of single-cell ERK-KTR N/C signals across a population of emitters in an all-emitter monolayer. Specific results were presented in Fig 2 and S2 Fig. (PDF) [file pcbi.1009873.s021.pdf]

S2 Text. Variability analysis of ERK-KTR N/C signal across emitters for individual all-emitter experiments

For all-emitter experiments, we wanted to better quantify the variability in the ERK-KTR N/C signal within cells across successive bPAC input pulses as well as between cells. To enable this, we calculated the mean and standard deviation, under different normalizations, for the ERK-KTR N/C signal for the cell population of the imaged monolayer. For a given normalization approach, each single cell ERK-KTR N/C signal is shifted such that the signal at the beginning of the first pulse is zero and then normalized. Mean and standard deviation are calculated to just before the start of the second pulse. This process is the then repeated for the second pulse enforcing the standard deviation is zero at the beginning of each pulse. For this discussion, we present data for all-emitter experiments for successive pulses of the same amplitude (Figs 2E and S2A) as well as successive pulses of increasing amplitude (Figs 2F and S2B).

For the first case, we plot the mean ± standard deviation of ERK-KTR N/C signal over the cell population for each experiment (Figs 2E-F and S2A-B, top plot). Here we observe significant variability that is similar across all experiments. This variability is similar to what is observed from other inhibitions on the ERK pathway (Regot et al., Cell (2014)).

Hypothesizing that this is due to variability in amplitude magnitude across cells, we calculated the mean ± standard deviation after each shifted ERK-KTR N/C signal was normalized by the difference between the peak value of the signal during the first pulse and the value at the beginning of the first pulse. The results are plotted in Figs 2E-F and S2A-B (second plot from top) where we see that the variability in the first pulse is greatly reduced from that in the first case (Figs 2E-F and S2A-B, top plot). This is because the amplitude scaling conditioned on the first pulse removes the effects of amplitude variability, especially in the first pulse, allowing for a better understanding of the behavior in the signal dynamics, we denote as time-dependent signal shape.

If the single cell signals in the first pulse all have the same shape but different amplitudes, then once amplitude is normalized out, they would all collapse onto each other during that first pulse and thus look identical. This is what we approximately observe in the first pulse, with some residual variability that is similar across all experiments. Thus our results, indicate that population variability is dominated by variability in the peak ERK-KTR N/C amplitude during the pulse.

However, the second pulse still has a lot of variability, less so for successive pulses of the same amplitude (Figs 2E-F and S2A-B, second plot from top). To determine if this is amplitude variability in the second pulse, for each cell, we applied the same prior normalization approach of the first pulse, but to the second pulse instead which removes the effects of peak amplitude variability in the second pulse. Indeed, the variability collapses in the second pulse and gets larger in the first (Figs 2E-F and S2A-B, third plot from top), indicating it is amplitude variability. One can see that conditioning for amplitude for each pulse, yields low residual variability, indicating that the time-dependent shape is quite similar between pulses as is observed in many of the single cells (S2A-B Fig, single cell plots).

Next we discuss the ERK-KTR response across successive bPAC input pulses within single cells and how this varies across the population. To begin, we observe that when the peak response in the first pulse is normalized, the variability in the second pulse is larger than the highly constrained variability of the first pulse (Figs 2E-F and S2A-B, second plot from top), less so for pulse sequences of the same amplitude. When the response in the second pulse is normalized, the variability in the first pulse is larger than the highly constrained variability during the second pulse (Figs 2E-F and S2A-B, third plot from top). For experiments with pulse sequences of the same amplitude, this implies that in single cells, the amplitude of the ERK-KTR N/C signal during the first and that during the second bPAC input pulse will be approximately the same for some of the cells, a little smaller during the second pulse for some cells, and a little bigger during the second pulse for some cells. For experiments with pulse sequences of increasing amplitude, these results imply that some cells respond proportionally (linearly) in their ERK-KTR N/C response to the input pulse amplitude, while some response less than proportionally, and others response more than proportionally (S1C Fig, single cell examples, from same experiment analyzed in Fig 2F). Furthermore, an approximately proportional response is also observed for experiments with multi-step inputs of increasing amplitude (S1D Fig, population average). This demonstrates that the proportional response occurs whether the pulses are spread out or touching (multi-step).

Overall, we observe that for the all-emitter monolayer, the time dependent ERK-KTR N/C response across cells follow a simple first-order like behavior upon bPAC activation at any amplitude we tested.
