# Supplementary material for: Analysis of localized cAMP perturbations within a tissue reveal the effects of a local, dynamic gap junction state on ERK signaling
Source: PLoS Comput Biol. 2022 Mar 30;18(3):e1009873. doi: 10.1371/journal.pcbi.1009873 (PMC9000136; doi:10.1371/journal.pcbi.1009873)
Supplement: S3 Text — This section discusses bPAC experiments with PKA and/or Epac inhibited and the resulting ERK-KTR dynamics. (PDF) [file pcbi.1009873.s022.pdf]

S3 Text. Both PKA and Epac contribute to the ERK-KTR N/C signal

While it is known that both Epac and PKA inhibit ERK [1-2], we wished to determine their relative contributions to this inhibition. To begin, for cells with constitutively active endogenous adenalyl cyclase, we wanted to observe the effects adding of Epac and/or PKA inhibitor to these cells thereby allowing us to gauge the level of decreased EKR-KTR N/C signal due to the inhibitor. To interrogate this experimentally, we performed all-receiver (no bPAC) experiments adding the adenalyl cyclase activator Forskolin at twenty minutes and then an inhibitor (Epac inhibitor (ESI-09, 15 μM, [3]), or PKA inhibitor (H89, 10 μM), or both) at forty minutes (S5A Fig), observing the same cells through the experiment. Both the Epac and PKA inhibitors and their combination have a similar effect in strongly reducing the ERK-KTR N/C signal. Thus, both PKA and Epac are strong contributors to the cAMP driven ERK-KTR N/C signal.

Next, we performed all-emitter experiments with a single pulse bPAC input sequence, then added an inhibitor (Epac inhibitor, or PKA inhibitor, or both), then repeated the bPAC input sequence, observing the same cells throughout the experiment (S5B Fig). Here we wanted to get a sense of the reduction in ERK-KTR N/C signal amplitude during the second bPAC input pulse (with inhibition) relative to the first (without inhibition). We observe that the reduction in ERK-KTR N/C signal under inhibition during the second pulse sequence is very similar for both inhibitors independently as well as for the combined inhibition. We should note that ESI-09 competes with cAMP to bind to Epac. Given that bPAC, for the chosen bPAC input amplitude, is producing a higher ERK-KTR N/C signal than Forskolin, i.e. more cAMP, we don't expect the percentage reduction in the ERK-KTR N/C signal to be as great for the bPAC case. This is what we observe.

Importantly, for an emitter in a small emitter cluster that shows overshoot during its first pulse (no drug), when PKA is inhibited before the second pulse, the emitter signal show little to no overshoot during the second pulse (Fig 4C) which is expected given that the gap-junctions are inhibited. This would not be expected under Epac inhibition since Epac is not implicated in the control of gap-junctions. Therefore, if we instead add Epac inhibitor before the second pulse, the emitter signal during the second pulse might still be expected to exhibit overshoot. This is what we observe (Fig 4D).

References

1. Nedvetsky PI, Kwon SH, Debnath J, Mostov KE. Cyclic AMP regulates formation of mammary epithelial acini in vitro. Mol Biol Cell. 2012;23(15):2973-2981.
2. Mizuno R, Kamioka Y, Kabashima K, Imajo M, Sumiyama K, Nakasho E, et al. In vivo imaging reveals PKA regulation of ERK activity during neutrophil recruitment to inflamed intestines. Journal of Experimental Medicine. 2014;211(6):1123-1136. doi:10.1084/jem.20132112.
3. Zhu Y, Chen H, Boulton S, Mei F, Ye N, Melacini G, Zhou J, Cheng X. Biochemical and pharmacological characterizations of ESI-09 based EPAC inhibitors: defining the ESI-09 "therapeutic window". Sci Rep, 5:9344, Mar 2015.
