# Supplementary material for: Analysis of localized cAMP perturbations within a tissue reveal the effects of a local, dynamic gap junction state on ERK signaling
Source: PLoS Comput Biol. 2022 Mar 30;18(3):e1009873. doi: 10.1371/journal.pcbi.1009873 (PMC9000136; doi:10.1371/journal.pcbi.1009873)
Supplement: S4 Text — This section goes through the assumptions, parameters and equations of the multicellular model. It also justifies the well-mixed assumption used. (PDF) [file pcbi.1009873.s023.pdf]

S4 Text. Details of multicellular model

In this section, we present the computational model developed to capture the salient features of the bPAC coupling to the cAMP/PKA and ERK pathways at both the intracellular and intercellular levels. The model we build results in a system of ordinary differential equations that is coded and simulated in MATLAB using the ODE solver ODE15s.

Model Variables

The variables in the model describe the number of molecules rather than concentrations. For ease of notation and clarity of model equations, we will put square brackets around each variable, e.g. $[X]$. A description of the model variables is given in the table below.

variable	description
$cAMP^i$	cAMP in cell i
PKA^i_{off}	inactive PKA in cell i
PKA^i_{on}	active PKA in cell i
PDE^i_{off}	low (basal) active state of PDE in cell i
PDE^i_{on}	hi active state of PDE in cell i
ERK^i_{off}	inactive state of ERK in cell i
ERK^i_{on}	active state of ERK in cell i
$EKTR^i_c$	cytosolic ERK-KTR in cell i
$EKTR^i_n$	nuclear ERK-KTR in cell i
GJ^i_{na}	inactive gap-junction molecule in cell i
$GJ^i_{d_n}$	gap-junction molecule, in cell i, in the nth intermediate state of the delayed gap-junction regulation model which has N total intermediate states.
GJC^i_a	active gap-junction complex (including Ezrin/PKA/connexin43) in cell i on cell-cell interface, initiated by the delayed gap-junction regulation model in cell i. The active gap-junction complex is also formed with connexin43 from the other cell at the interface to create a working gap-junction.

input	description, all inputs can take on values between 0 and 1
$I_{bPAC}(t)$	modulates the cAMP production rate through blue light induced bPAC
$I_{IBMX}(t)$	inhibits PDE via IBMX

Model Assumptions

In building the computational model, we adopted the following biologically motivated assumptions:

- Each cell has a basal production of cAMP. Cells that contain bPAC can produce cAMP through light-activated bPAC.
- All cells have the same volume.
- cAMP can be tranported to neighboring cells through gap junctions that are in the activated state (positively regulated through cAMP/PKA).
- Our model assumes that cAMP is well-mixed (and other species as well), i.e. spatially uniform concentration (approximately) within each cell. In addition, experimentally, bPAC is cytosolic and likely spatially uniform. Thus, there is a spatially uniform source of cAMP. The well-mixed assumption allows us not to have to use a computationally intensive reaction-diffusion model within each cell. For a more in-depth biophysical discussion of the well-mixed assumption see section "Biophysical arguments for the well-mixed assumption" at the bottom.
- For cells that are coupled through gap-junctions, the net rate of flux of cAMP from cell i to cell j uses a model of the form $\rho_{ij} \times \left([cAMP^j] - [cAMP^i]\right)$, i.e., the effective gap-junctional cAMP permeability (can be time-dependent) times the concentration difference between the two cells [1-4].

- For the active gap-junction complex, GJC_a^i, initiated in cell i, we assume that other cell at the cell-cell interface provides connexin43 to enable the formation of the complete active gap-junction complex, GJC_a^i (allowing cell-cell coupling of cAMP through it). This assumption is made to keep the model simple.

Below we present two multicellular models. The first is the Minimal (M) Model. This is the general model used for all results except those presented in Figs 5H and S6H-I which use the second model, the Intracellular Overshoot (IO) model. The organization of the presentation begins with the description of the reactions and their parameters that appear in the M model and/or the IO model. We then present the kinetic equations of the M model and then that for the IO model.

Discussion of parameters for the propensity term for each reaction for the multicellular system used by the Minimal (M) and/or the Intracellular Overshoot (IO) models

Reaction 1: $\emptyset \xrightarrow{c_b^i I_{bPAC}(t)\beta_{c,b}} cAMP^i$. This chemical equation describes the bPAC production of cAMP with propensity $c_b^i I_{bPAC}(t)\beta_{c,b}$ where:

c_b^i - is 1 if cell i contains bPAC (Emitter (E)), otherwise 0 (Receiver (R)).

$\beta_{c,b}$ - max creation rate of cAMP through bPAC. Here we limit the bPAC input $I_{bPAC}(t)$ to be between zero and one.

models - M and IO.

Reaction 2: $\emptyset \xrightarrow{\beta_{c,ac}\frac{X_{f_1}}{c_{ac,pk}[PKA_{on}^i]+X_{f_1}}} cAMP^i$. This chemical equation describe the production of cAMP through endogenous adenalyl cyclase with propensity term $\beta_{c,ac}\frac{X_{f_1}}{c_{ac,pk}[PKA_{on}^i]+X_{f_1}}$ where:

$\beta_{c,ac}$ - max creation rate of cAMP through endogenous adenalyl cyclase negatively regulated through PKA via the function $\frac{X_{f_1}}{c_{ac,pk}[PKA_{on}^i]+X_{f_1}}$.

X_{f_1} - the IC50 value for the PKA-dependent negative feedback function on the endogenous adenalyl cyclase.

$c_{ac,pk}$ - can be 0 or 1. When $c_{ac,pk} = 1$, there is negative feedback. When $c_{ac,pk} = 0$ there is just a constant creation rate.

models - M and IO.

Reaction 3: $cAMP^i \xrightarrow{\gamma_c[cAMP^i]} \emptyset$. This chemical equation describes the basal non-PDE degradation of cAMP with propensity term $\gamma_c[cAMP^i]$. where:

γ_c - degradation rate of cAMP through basal non-PDE activity.

models - M and IO.

Reaction 4: $cAMP^i \xrightarrow{\gamma_{c,pd_b}[cAMP^i]} \emptyset$. This chemical equation describes the basal PDE degradation of cAMP with propensity term $\gamma_{c,pd_b}[cAMP^i]$ where:

γ_{c,pd_b} - degradation rate of cAMP through basal PDE activity.

models - M and IO.

Reaction 5: $cAMP^i \xrightarrow{\gamma_{c,pd2}[cAMP^i]\frac{[PDE_{on}^i]}{[PDE_{on}^i]+X_{f_2}}} \emptyset$. This chemical equation describes the PDE dependent degradation of cAMP with propensity term $\gamma_{c,pd2}[cAMP^i]\frac{[PDE_{on}^i]}{[PDE_{on}^i]+X_{f_2}}$ where:

$\gamma_{c,pd2}$ - max degradation rate of cAMP through PKA regulated PDE activity.

X_{f_2} - is the EC50 value of the positive PDE regulated hill function $\frac{[PDE_{on}^i]}{[PDE_{on}^i]+X_{f_2}}$. PKA positively regulates PDE (see reaction 11 below).

models - IO.

Reaction 6: $cAMP^i \xrightarrow{\omega_{ij}k_{gj}[cAMP^i]} cAMP^j$. This chemical equation describes the flux of cAMP from cell i to cell j through ij coupled gap-junctions with propensity $\omega_{ij}k_{gj}[cAMP^i]$ where:

k_{gj} - the basal permeability of cAMP transport through gap-junctions between a given cell i and cell j pair if $\omega_{ij} = 1$. ω_{ij} is the the connection constant between cell i and cell j where for zero they are not connected and for one, they are. And where $\omega_{ij} = \omega_{ji}$, i.e. either cell can transport cAMP to the other. Fig 5F and S7C Fig each show a particular configuration of connected cells.

models - M and IO.

Reaction 7: $cAMP^i \xrightarrow{\omega_{ij}\frac{k_{gj,gjf}\bar{P}}{X_{f_3}}\big(\frac{[GJC_a^i]}{P_i}+\frac{[GJC_a^j]}{P_j}\big)[cAMP^i]} cAMP^j$. This chemical equation describes the flux of cAMP from cell i to cell j, for the delayed gap-junction regulation model, through ij coupled gap-junctions with propensity $\omega_{ij}\frac{k_{gj,gjf}\bar{P}}{X_{f_3}}\big(\frac{[GJC_a^i]}{P_i}+\frac{[GJC_a^j]}{P_j}\big)[cAMP^i]$ where ω_{ij} is the same as in Reaction 6 and where:

$k_{gj,gjf}$ - the max permeability of cAMP transport through gap-junctions between a given cell i and cell j pair regulated by the delayed gap-junction regulation model, if $\omega_{ij} = 1$.

P_i, P_j - P_i is the number of cells that cell i couples to and P_j is the number of cells that cell j couples to. Within each cell, the active gap-junction concentration is parsed equally to each cell they couple to, hence $\frac{[GJC_a^i]}{P_i}+\frac{[GJC_a^j]}{P_j}$ are the parsed concentrations from cells i and j, on the ij interface.

X_{f_3} - is the total pool of gap-junctions that can be activated. Thus, $\frac{1}{X_{f_3}}\big(\frac{[GJC_a^i]}{P_i}+\frac{[GJC_a^j]}{P_j}\big)$ represents the fraction of activated gap junctions at each interface through bPAC induction of cAMP/PKA. The $\frac{[GJC_a^i]}{P_i}+\frac{[GJC_a^j]}{P_j}$ term ensures that the i and j cells have equal permeability to transport cAMP to the other cell.

$\bar{P}$ - The term $\bar{P}$ is the average number of cells that the cells couple to. For the paper, $\bar{P} = 5$, where most P_i typically vary between 4 and 7. $\bar{P}$ and X_{f_3} and are normalizing constants so that $k_{gj,gjf}$ has the same dimensions as the basal term k_{gj}.

models - M.

Reaction 8: $PKA_{off}^i \xrightarrow{k_{pk,c}[PKA_{off}^i]\frac{[cAMP^i]^{n_{f_5}}}{[cAMP^i]^{n_{f_5}}+X_{f_5}^{n_{f_5}}}} PKA_{on}^i$. This chemical equation describes the activation of PKA through cAMP with propensity $k_{pk,c}[PKA_{off}^i]\frac{[cAMP^i]^{n_{f_5}}}{[cAMP^i]^{n_{f_5}}+X_{f_5}^{n_{f_5}}}$ where:

$k_{pk,c}$ - activation rate of PKA regulated through cAMP.

X_{f_5} - EC50 of positive cAMP dependent hill function.

n_{f_5} - Hill coefficient of positive cAMP dependent hill function.

models - M and IO.

Reaction 9: $PKA_{on}^i \xrightarrow{\gamma_{pk}[PKA_{on}^i]} PKA_{off}^i$. This chemical equation describes the deactivation of PKA with propensity $\gamma_{pk}[PKA_{on}^i]$ where:

γ_{pk} - PKA deactivation rate.

models - M and IO.

Reaction 10: $PKA_{on}^i \xrightarrow{\gamma_{pk,pd}[PKA_{on}^i]\frac{[PDE_{on}^i]}{[PDE_{on}^i]+X_{f_4}}} PKA_{off}^i$. This chemical equation describes the deactivation of PKA through PDE feedback with propensity $\gamma_{pk,pd}[PKA_{on}^i]\frac{[PDE_{on}^i]}{[PDE_{on}^i]+X_{f_4}}$ where:

$\gamma_{pk,pd}$ - PKA deactivation rate for the PDE-dependent negative feedback.

X_{f_4} - EC50 value for the PDE-dependent negative feedback function.

models - IO.

Reaction 11: $PDE_{off}^i \xrightarrow{k_{pd,pk}[PDE_{off}^i][PKA_{on}^i]} PDE_{on}^i$. This chemical equation describes the activation of PDE through PKA with propensity $k_{pd,pk}[PDE_{off}^i][PKA_{on}^i]$ where:

$k_{pd,pk}$ - PDE activation rate through PKA.

models - IO.

Reaction 12: $PDE_{on}^i \xrightarrow{k_{pd}[PDE_{on}^i]} PDE_{off}^i$. This chemical equation describes the deactivation of PDE with propensity $\gamma_{pd}[PDE_{on}^i]$ where:

γ_{pd} - deactivation rate of PDE.

models - IO.

Reaction 13: $ERK_{off}^i \xrightarrow{k_e} ERK_{on}^i$. This chemical equation describes the activation of ERK with propensity k_e where:

k_e - basal activation rate of ERK.

models - M and IO.

Reaction 14: $ERK_{on}^i \xrightarrow{\gamma_e[ERK_{on}^i]} ERK_{off}^i$. This chemical equation describes the deactivation of ERK with propensity $\gamma_e[ERK_{on}^i]$ where:

γ_e - deactivation rate of ERK.
models - M and IO.

Reaction 15: $ERK^i_{on} \xrightarrow{\gamma_{e,c}[ERK^i_{on}]\frac{[cAMP^i]}{[cAMP^i]+X_{f_6}}} ERK^i_{off}$. This chemical equation describes the cAMP-dependent deactivation of ERK with propensity $\gamma_{e,c}[ERK^i_{on}]\frac{[cAMP^i]}{[cAMP^i]+X_{f_6}}$ where:

$\gamma_{e,c}$ - deactivation rate of ERK through cAMP-dependent deactivation.
X_{f_6} - EC50 value for cAMP-dependent positive hill function.
models - M and IO.

Reaction 16: $EKTR^i_c \xrightarrow{\gamma_{ek}[EKTR^i_c]} EKTR^i_n$. This chemical equation describes the deactivation of ERK-KTR to nucleus with propensity $\gamma_{ek}[EKTR^i_c]$ where:

γ_{ek} - deactivation/nuclear localization rate of cytosolic ERK-KTR.
models - M and IO.

Reaction 17: $EKTR^i_n \xrightarrow{k_{ek,e}[ERK^i_{on}][EKTR^i_n]} EKTR^i_c$. This chemical equation describes the ERK dependent activation of ERK-KTR to cytosol with propensity $k_{ek,e}[ERK^i_{on}][EKTR^i_n]$ where:

$k_{ek,e}$ - activation/cytosolic localization rate of EKR-KTR through ERK.
models - M and IO.

Reaction 18: $GJ^i_{na} \xrightarrow{\beta_{gj,pk}[GJ^i_{na}]\frac{[PKA^i_{on}]}{[PKA^i_{on}]+X_{f_7}}} GJ^i_{d_1}$. This chemical equation describes the transition of inactive GJ to first intermediate state of the delayed gap-junction regulation model with propensity $\beta_{gj,pk}[GJ^i_{na}]\frac{[PKA^i_{on}]}{[PKA^i_{on}]+X_{f_7}}$ where:

$\beta_{gj,pk}$ - transition rate of inactive GJ to first intermediate state of the delayed gap-junction regulation model through PKA.
X_{f_7} - EC50 value of the PKA-dependent positive hill function.
models - M.

Reaction 19: $GJ^i_{d_{n-1}} \xrightarrow{\frac{N}{\tau_{gj}}[GJ^i_{d_{n-1}}]} GJ^i_{d_n}$. This chemical equation describes the transition between intermediate states of the delayed gap-junction regulation model ($n = 2$ to N) with propensity $\frac{N}{\tau_{gj}}[GJ^i_{d_{n-1}}]$ where:

N - number of intermediate states between inactive GJ and active GJC for the delayed gap-junction regulation model.
τ_{gj} average time to go from first activating intermediate state to active GJC.
models - M.

Reaction 20: $GJ^i_{d_N} \xrightarrow{\frac{N}{\tau_{gj}}[GJ^i_{d_N}]} GJC^i_a$. This chemical equation describes the transition from last intermediate state of the delayed gap-junction regulation model to active GJC with propensity $\frac{N}{\tau_{gj}}[GJ^i_{d_N}]$ with same parameters as the propensity in Reaction 19 where:

models - M.

Reaction 21: $GJC^i_a \xrightarrow{\gamma_{gj}[GJC^i_a]} GJ^i_{na}$. This chemical equation describes the transition from active GJC to inactive GJ with propensity $\gamma_{gj}[GJC^i_a]$ where:

γ_{gj} - deactivation rate of active GJC.
models - M.

Kinetic Multicellular Model Equations for the Minimal (M) Model, i.e. no feedback on PDEs

The Minimal Model is the general model used for all results except those presented in Figs 5H and S6H-I which use the Intracellular Overshoot (IO) model.

Description of Minimal Model Equations:

- The time-dependent $[cAMP^i]$ equation uses reactions 1, 2 , 3, 4, 6, and 7. For cAMP degradation there are no feedbacks on cAMP degradation, only a non-PDE degradation term (reaction 3) and a basal PDE-dependent degradation term (reaction 4) that gets shutoff when IBMX is added. Both the basal gap-junction flux model (reaction 6) and the delayed gap-junction regulation flux model (reaction 7) are used.

- The time-dependent $[PKA^i_{off}]$ equation uses reactions 8 and 9.
- The time-dependent $[ERK^i_{off}]$ equation uses reactions 13, 14, and 15.
- The time-dependent $[EKTR^i_n]$ equation uses reactions 16 and 17.
- The time-dependent equations for the delayed gap-junction regulation model use reactions 18, 19, 20 and 21.

Minimal Model Equations:

$$
\begin{aligned}
\frac{d[cAMP^i]}{dt} &= I_{bPAC}(t)\beta_{c,b} + \beta_{c,ac}\frac{X_{f_1}}{c_{ac,pk}[PKA^i_{on}] + X_{f_1}} - \gamma_c[cAMP^i] - (1 - I_{IBMX}(t))\gamma_{c,pd_b}[cAMP^i] \\
&\quad + \sum_{j\neq i}\omega_{ij}\left[k_{gj} + \frac{k_{gj,gjf}\bar{P}}{X_{f_3}}\left(\frac{[GJC^i_a]}{P_i} + \frac{[GJC^j_a]}{P_j}\right)\right]([cAMP^j] - [cAMP^i]) \\
\frac{d[PKA^i_{off}]}{dt} &= \gamma_{pk}[PKA^i_{on}] - k_{pk,c}[PKA^i_{off}]\frac{[cAMP^i]^{n_{f_5}}}{[cAMP^i]^{n_{f_5}} + X_{f_5}^{n_{f_5}}} \\
\frac{d[ERK^i_{off}]}{dt} &= \gamma_e[ERK^i_{on}] + \gamma_{e,c}[ERK^i_{on}]\frac{[cAMP^i]}{[cAMP^i] + X_{f_6}} - k_e[ERK^i_{off}] \\
\frac{d[EKTR^i_n]}{dt} &= \gamma_{ek}[ERK^i_c] - k_{ek,e}[ERK^i_{on}][EKTR^i_n] \\
\frac{d[GJ^i_{na}]}{dt} &= -\beta_{gj,pk}[GJ^i_{na}]\frac{[PKA^i_{on}]}{[PKA^i_{on}] + X_{f_7}} + \gamma_{gj}[GJC^i_a] \\
\frac{d[GJ^i_{d_1}]}{dt} &= \beta_{gj,pk}[GJ^i_{na}]\frac{[PKA^i_{on}]}{[PKA^i_{on}] + X_{f_7}} - \frac{N}{\tau_{gj}}[GJ^i_{d_1}] \\
\frac{d[GJ^i_{d_n}]}{dt} &= \frac{N}{\tau_{gj}}[GJ^i_{d_{n-1}}] - \frac{N}{\tau_{gj}}[GJ^i_{d_n}] \\
\frac{d[GJC^i_a]}{dt} &= \frac{N}{\tau_{gj}}[GJ^i_{d_N}] - \gamma_{gj}[GJC^i_a]
\end{aligned}
$$

with $\frac{d[PKA^i_{off}]}{dt} = -\frac{d[PKA^i_{on}]}{dt}$, $\frac{d[ERK^i_{off}]}{dt} = -\frac{d[ERK^i_{on}]}{dt}$, and $\frac{d[EKTR^i_n]}{dt} = -\frac{d[EKTR^i_c]}{dt}$. Equations are solved in MATLAB using the ordinary differential equation solver ODE15s.

Kinetic equations for the Intracellular Overshoot (IO) model whose intracellular circuit system produces overshoot in the cAMP and ERK-KTR N/C signals, even in the absence of cell-cell coupling
The Intracellular Overshoot (IO) is used for the results in Figs 5H and S6H-I.

Description of Intracellular Overshoot (IO) Model Equations:

- The time-dependent $[cAMP^i]$ equation uses reactions 1, 2, 3, 4, 5, and 6. For cAMP degradation is a non-PDE degradation term (reaction 3) and a basal PDE-dependent degradation term (reaction 4) that gets shutoff when IBMX is added. In addition, there is a degradation term with explicit PDE dependence through a feedback from PKA (reaction 4) which gets shutoff when IBMX is added. Only the basal gap-junction flux model (reaction 6) is used.
- The time-dependent $[PKA^i_{off}]$ equation uses reactions 8 and 9. It also uses reaction 10, deactivation of PKA through a PDE feedback.
- The time-dependent $[PDE^i_{off}]$ equation uses reactions 11 and 12 where reaction 12 is PDE activation through PKA.
- The time-dependent $[ERK^i_{off}]$ equation uses reactions 13, 14, and 15.
- The time-dependent $[EKTR^i_n]$ equation uses reactions 16 and 17.

Intracellular Overshoot Model Equations:

$$\begin{aligned}
\frac{d[cAMP^i]}{dt} &= c_b^i I_{bPAC}(t)\beta_{c,b} + \beta_{c,ac}\frac{X_{f_1}}{[PKA^i_{on}] + X_{f_1}} - \gamma_c cAMP \\
&\quad -(1 - I_{IBMX}(t))\gamma_{c,pd_b} cAMP - (1 - I_{IBMX}(t))\gamma_{c,pd2}[cAMP^i]\frac{[PDE^i_{on}]}{[PDE^i_{on}] + X_{f_2}} \\
&\quad + \sum_{j\neq i}\omega_{ij}k_{gj}\big([cAMP^j] - [cAMP^i]\big) \\
\frac{d[PKA^i_{off}]}{dt} &= \gamma_{pk}[PKA^i_{on}] + \gamma_{pk,pd}[PKA^i_{on}]\frac{[PDE^i_{on}]}{[PDE^i_{on}] + X_{f_4}} \\
&\quad -k_{pk,c}[PKA^i_{off}]\frac{[cAMP^i]^{n_{f5}}}{[cAMP^i]^{n_{f5}} + X_{f_5}^{n_{f5}}} \\
\frac{d[PDE^i_{off}]}{dt} &= \gamma_{pd}[PDE^i_{on}] - k_{pd,pk}[PDE^i_{off}][PKA^i_{on}] \\
\frac{d[ERK^i_{off}]}{dt} &= \gamma_e[ERK^i_{on}] + \gamma_{e,c}[ERK^i_{on}]\frac{[cAMP^i]}{[cAMP^i] + X_{f_6}} - k_e[ERK^i_{off}] \\
\frac{d[EKTR^i_n]}{dt} &= \gamma_{ek}[ERK^i_c] - k_{ek,e}[ERK^i_{on}][EKTR^i_n]
\end{aligned}$$

with $\frac{d[PKA^i_{off}]}{dt} = -\frac{d[PKA^i_{on}]}{dt}$, $\frac{d[PDE^i_{off}]}{dt} = -\frac{d[PDE^i_{on}]}{dt}$, $\frac{d[ERK^i_{off}]}{dt} = -\frac{d[ERK^i_{on}]}{dt}$, and $\frac{d[EKTR^i_n]}{dt} = -\frac{d[EKTR^i_c]}{dt}$. Equations are solved in MATLAB using the ordinary differential equation solver ODE15s.

Pathway proteins whose total number of molecules per cell (active + inactive states) remains constant during simulation

$[PKA_{tot}]$ - total PKA: 10 s-molecules/cell. (models: M and IO)
$[PDE_{tot}]$ - total PDE: 1 s-molecules/cell (models: IO)
$[ERK_{tot}]$ - total ERK: 10 s-molecules/cell (models: M and IO)
$[EKTR_{tot}]$ - total ERK-KTR: 10 s-molecules/cell (models: M and IO)
$[GJC_{tot}]$ - total GJC: 10 s-molecules/cell (models: M)

Here the 's' in s-molecules is a scaling, e.g. s corresponding to kilo would be kilo-molecules, for example. However, we keep it generic.

Biophysical arguments for the well-mixed assumption

Our model assumes that cAMP is well mixed, i.e. spatially uniform concentration (approximately), within each cell (compartment). In addition, experimentally, bPAC in emitter cells is cytosolic and not recruited to membranes and is therefore spatially uniform (approximately). Thus, there is a spatially uniform source of cAMP in emitters. Therefore, for the well-mixed assumption to hold, we need to show that the effective cAMP diffusion rate across the cell is much larger than the gap junction flux rate. In other words, the cAMP intracellular spread time is much faster than the exit time through gap-junctions. When this holds, the system can be considered well mixed since the intracellular concentration can spatially equilibrate much faster than molecules are exiting the cell, thus the concentration will be approximately uniform across the cell for both emitters and receivers.

In [2], based on the arguments above, the authors derive an expression for the well-mixed approximation, i.e., $p_0 L/D_{eff} \ll 1$ where p_0 is the gap-junction permeability at cell-cell interfaces, L is the cell length (for this paper we have circular(ish) cells to square(ish) cells), and D_{eff} is the effective diffusion coefficient. The authors derivation was for 1D, i.e. $n = 1$, where n is the dimension of the diffusion process. However for this paper, $n = 2$ for a 2D monolayer. Importantly, the dimension affects how a distribution of molecules will spread as a function of time. Specifically, for a group of localized cAMP molecules starting at time $t = 0$ their the spatial distribution

width σ as a function of time is $\sigma = \sqrt{2nD_{eff}t}$. Here D_{eff} is multiplied by n, thus n scales the effect of D_{eff}. We therefore adapted their expression to be

$$\frac{\frac{\bar{P}}{2}p_0 L}{nD_{eff}} \ll 1 \tag{s1}$$

and where we have added the term $\frac{\bar{P}}{2}$. Here $\bar{P}$ is the mean number of cell-cell interfaces. For the 1D system in [2], $\bar{P} = 2$ (each cell has a neighbor cell on each side (1D line of cells)), thus $\frac{\bar{P}}{2} = 1$. However, the more cell-cell interfaces a given cell has, the more flux through gap-junctions that can occur. For this paper, we set $\bar{P} = 5$. To evaluate if our system satisfies Eq. (s1), we must first get values for the parameters in the expression.

We first calculate the maximum permeability, p_0, at each cell-cell interface. From our multicellular model, $p_0 \approx k_{gj} + \frac{k_{gj,gjf}\bar{P}}{X_{f_3}} \max\left(\frac{[GJC_a^i]}{P_i} + \frac{[GJC_a^j]}{P_j}\right)$. From our simulations, we observe $\max\left(\frac{[GJC_a^i]}{P_i} + \frac{[GJC_a^j]}{P_j}\right) \leq .02$ at an emitter/reciever interface, and this value decreases the further a cell is away from the emitter. From our simulations $k_{gj} = .0104$, $k_{gj,gjf} = .2083$, X_{f_3}, and $\bar{P} = 5$. Therefore $p_0 \approx .0104 + .2083 * 5 * .02 = .0312$.

Next we researched D_{eff}. Recent work [5] measured the effective cAMP cytoplasmic diffusion coefficient in Ventricular myocytes (not MDCK cells, but we'll assume similar conditions) to be around $D_{eff} = 10\ \mu\text{m}^2/\text{s}$. This relatively slow diffusion coefficient includes the effects of binding events (e.g., to PKA), since a molecule of similar size that does not bind to other molecules has a measured diffusion coefficient of around 65 $\mu\text{m}^2/\text{s}$.

Finally, the MDCK cells in our experiments typically have a cell length of 20-30 μm, i.e. $L \leq 30\ \mu$m. Plugging our values for p_0, L and D_{eff}, we get $\frac{\frac{\bar{P}}{2}p_0 L}{nD_{eff}} = (5/2) * .0312 * 30/(2 * 10) = .11 << 1$, i.e. about an order of magnitude smaller than one. This is an upper limit based on the peak gap-junction activity we observe at the emitter/receiver interface (in the model) and for the largest cells we observe (in the experiments). Many cells are half this size. This result, valid for both emitters and receivers, supports our well-mixed assumption. While a full intracellular reaction-diffusion model might provide moderately higher accuracy [1-2], it should have minimal effect on the qualitative results such as modeling overshoot in single-emitter-cluster experiments.

References

1. Nitsche JM, Chang HC, Weber PA, Nicholson BJ. A transient diffusion model yields unitary gap junctional permeabilities from images of cell-to-cell fluorescent dye transfer between Xenopus oocytes. Biophys J. 2004;86(4):2058-2077.
2. Hofer T, Politi A, Heinrich R. Intercellular Ca2+ wave propagation through gap-junctional Ca2+ diffusion: a theoretical study. Biophys J. 2001;80(1):75-87.
3. De Blasio BF, Iversen JG, Røttingen JA. Intercellular calcium signalling in cultured renal epithelia: a theoretical study of synchronization mode and pacemaker activity. Eur. Biophys. J., 33(8):657-670, Dec 2004.
4. Benninger RK, Zhang M, Head WS, Satin LS, Piston DW. Gap junction coupling and calcium waves in the pancreatic islet. Biophys. J., 95(11):5048-5061, Dec 2008.
5. Agarwal SR, Clancy CE, Harvey RD. Mechanisms Restricting Diffusion of Intracellular cAMP. Sci Rep, 6:19577, Jan 2016.
