# Supplementary material for: Analysis of localized cAMP perturbations within a tissue reveal the effects of a local, dynamic gap junction state on ERK signaling
Source: PLoS Comput Biol. 2022 Mar 30;18(3):e1009873. doi: 10.1371/journal.pcbi.1009873 (PMC9000136; doi:10.1371/journal.pcbi.1009873)
Supplement: S5 Text — This section discusses the mobility phenotype under different pharmacological and genetic (emitter or receiver) perturbations. It goes into a more detailed and deeper discussion than the summary at the end of the Discussion section. (PDF) [file pcbi.1009873.s024.pdf]

S5 Text. bPAC induced cAMP in small emitter clusters is causal to a mobility phenotype in receivers

Unexpectedly, for small-emitter-cluster and single-emitter experiments, localized perturbations of cAMP through bPAC occasionally coincided with in an emergent mobility phenotype. Differential mobility in subgroups of receiver cells relative to small emitter clusters is observed (S11A, E, and F Fig). The mobility phenotype occurs near the beginning of the experiment from S11A Fig and about half-through the first pulse for the experiments in S11E-F Fig.

Table S1 presents different monolayer mosaics, e.g., small-emitter-clusters monolayers and all-emitter monolayers, and conditions under which we either observe a mobility phenotype, no mobility phenotype, or rarely observed it. For a given monolayer mosaic and condition, we list if cell-cell coupling is operational (or inhibited), and the number of experiments for which the mobility phenotype is observed out of the total (experimental frequency). It also breaks down a group of experiments into mobility or no mobility, and whether or not there was a search-induced ERK-KTR N/C signal (bPAC induced) at time t=0 (this occurs from exciting bPAC when searching for suitable cells to image with light that overlaps the spectrum that excites bPAC). It further breaks down the mobility groups into when the mobility started (early, during the first pulse, or mid-to-late in the experiment). Our aim for this presentation approach of the data is to have a relatively thorough set of monolayer mosaic types, pharmacological perturbations, and initial ERK-KTR N/C conditions to potentially constrain the requirements for the mobility phenotype. We also filtered out experiments where the cells were either all drifting in a particular direction or where the cells had a lot of random 'rearrangement' movement.

From Table S1, there are a number of small-emitter-cluster experiments that exhibited the mobility phenotype whose mobility initiated anywhere after the start of the first bPAC input pulse to the end of the second bPAC input pulse. This suggests that bPAC induction is potentially required (causal) for the observed mobility. However, there are also a number of experiments that exhibit the mobility phenotype early, i.e. before the first bPAC pulse, including the example from S11A Fig. For these, it is possible that bPAC induction is not causal. However, we wanted to see if those experiments had perturbed levels of ERK-KTR N/C signal in emitters at time $t = 0$ (i.e., the search-induced ERK-KTR N/C signal discussed above) which decays to baseline before the first pulse (see single cell examples in Fig 2B). Indeed the majority of experiments that observed mobility early in the experiment exhibited perturbed ERK-KTR N/C signals at $t = 0$, suggesting the bPAC induction might be causal for the these early mobility cases as well. This analysis includes experiments with small emitter clusters surrounded by either receiver or receivers-CX43-NGFP (receivers with over expressed connexin43 tagged with GFP at its N termius, this connexin43 forms a broken gap junction). Overall, these two groups have the highest experimental frequency of the mobility phenotype.

For small emitter clusters surrounded by receivers under PKA inhibition (S11D Fig) or gap junction inhibition (S11C Fig), no mobility phenotypes are observed. For small emitter clusters surrounded by receivers-MDCKII cells (receivers that do not form gap junctions) the experimental frequency is low with only two cases exhibiting the mobility phenotype, but both with perturbed levels of ERK-KTR N/C signal in emitters at time $t = 0$ and none after the beginning of the first pulse.

From Table S1, the mobility phenotype does not occur in an all-emitter experiment (example in S11B Fig) except for one case under PKA inhibition with no perturbed levels of ERK-KTR N/C signal in emitters at time $t = 0$. This one case is likely a coincidence of a small group of mobile cells at the beginning of the experiment, given that all cells are emitters.

Overall, the data suggests that the mobility phenotype requires a cluster of emitters surround by receivers. It also requires cAMP, and PKA and potentially the formation of emitter/receiver gap junctions given that the small emitter cluster surrounded by receiver-MDCKII cells has low experimental frequency.

The most striking mobility phenotype has been observed in the experiments with small emitter cluster surrounded by receiver-CX43-NGFP cells (S11E Fig, for example (see S5 Movie with description)). Due to the broken gap-junctions form by the connexin43-NGFP in the receivers (see upper illustrations in S8B Fig), there is reduced emitter/receiver cAMP coupling and, thus, a large differential in cAMP and PKA activity between the emitter and receiver-CX43-NGFP cells. This differential is likely much larger than that between emitter and receiver cells in normal emitter/receiver experiments. This potentially points to a strong PKA activity gradient between emitter and receiver-CX43-NGFP cells that is somehow sensed, perhaps mechanically. Importantly, not all receivers become mobile, just subgroups of them, suggesting a local group decision.

A question arises: would the mobility phenotype also occur if, for a localized group of cells, we activated G-protein coupled receptors (GPCRs) which up-regulates cAMP, but also interact with other signal molecules? Or might the mobility phenotype be inhibited through the other interactions of the GPCR? Either way, it would be interesting to find out.
