# Supplementary material for: Analysis of localized cAMP perturbations within a tissue reveal the effects of a local, dynamic gap junction state on ERK signaling
Source: PLoS Comput Biol. 2022 Mar 30;18(3):e1009873. doi: 10.1371/journal.pcbi.1009873 (PMC9000136; doi:10.1371/journal.pcbi.1009873)
Supplement: Attachment — Submitted filename: cover_letter_and_detailed_response.pdf [file pcbi.1009873.s025.pdf]

UCSF
University of California
San Francisco

Michael Chevalier, Ph.D.
Associate Professional Researcher
Department of Biochemistry & Biophysics

1700 4th St,, MC2542
San Francisco, CA 94158
tel: 650-521-2851
email: mikchevy@alumni.stanford.edu

December 20, 2021

Dear Dr. Meier-Schellersheim and Dr. Haugh,

I am writing to submit our revised manuscript entitled "Analysis of localized cAMP perturbations within a tissue reveal the effects of a local, dynamic gap junction state on ERK signaling" for possible publication in PLOS Computational Biology as a Research Article.

We appreciate the reviewers' efforts and their resulting comments. They have helped us to strengthen the paper. Our response to the reviewers' comments is detailed in the pages below.

Again, thank you for the opportunity to revise the paper. We are truly excited by the prospect of publishing our work in your journal.

Sincerely yours,

Michael Chevalier, PhD

Response to Reviewers

December 20, 2021

Response to Reviewer 1

Reviewer 1: Review Comments to the Authors: In the present work entitled: Analysis of localized cAMP perturbations within a tissue reveal the effects of a local, dynamic gap junction state on ERK signaling, Fonseca J. and colleagues showed by a combination of pharmacological, imaging and computational modeling approaches that cAMP production crosses gap junction in epithelial cells to inhibit ERK signaling. Authors performed complementary experiments at cellular level using pertinent emitter cell model of bPAC with receiver cells expressing signaling pathway reporters. First, this study, describes their study models and relationships between bPAC induced emitters (in small and single emitter cluster) with ERK-KTR in emitters and receivers. Next, authors performed empirical studies to fit their experimental observations with an appropriate computational model. Overall, we found this study to be well written although the topic and conclusion might seem narrowed. Some experiments and associated conclusions are clearly convincing, some, however, need additional controls or re-analysis. Therefore, we have comments to address to authors, which would strengthen the paper and conclusions.

Authors: We thank the reviewer, Dr. Pidoux, for reviewing our manuscript. Given his expertise in PKA mediated control of gap-junctions, we are grateful for his insights and opinions. We have taken his comments to heart and have done our best to address them. We have adjusted figures and text. We'll now go through the comments.

Reviewer 1, Major comment 1: Authors provided an extensive work here with a large amount of analysis. However, this large amount of information often makes the work difficult to follow. Therefore, the rational between experiment/data are often confused and unclear that would need explanation/introduction. Furthermore, some supplementary data do not provide additional information to the story and should be removed. Also (but not only), Fig S5 looks to us more pertinent to show than some panels in Fig 4 (ie PKA and Epac). Therefore, we recommend to authors to reorganize the paper (text and figure) to optimize their message (somewhat important) and increase readability.

Authors: We appreciate the reviewer's thoughts on developing a clearer narrative and presentation throughout the paper. One important thing we did was break up the discussion of Figure 3 into more subsections with subsection titles so that the reader goes into a subsection knowing what to expect. And within each subsection we have added more introduction and motivation.

For Figure 4, we have taken the reviewers suggestion and have moved the small-emitter experiments from Fig S5C (PKA inhibitior added halfway through) and Fig S5D (Epac inhibitior added halfway through) to Figure 4C and 4D. This helps make Figure 4 more complete. We appreciate this suggestion.

Updates of Figure 3 and 4 legends are in red. And updates of the referencing to Figure 4 is in the main text on page 10, tagged with R1MC1)

Reviewer 1, Major comment 2: We believe that some additional control would support their conclusion. Hence, does authors would observe similar data after uncaging cAMP (instead of bPAC)? This would support their observation and validate somehow the proposed mathematical model. Does the illumination used to stimulate cAMP production affects ERK-KTR in receiver cells lacking bPAC? Does blue pulse affects directly gap junction communication?

Authors: We'll address the latter comments first. We do have data showing that pulsed blue-light illumination of receiver cells does not induce an ERK-KTR N/C signal. We have placed this in Figure S1G with discussion in Text S1 at the end of the second to last paragraph (red text tagged with R1MC2).

The question as to whether or not the blue light pulse directly affects gap junction communication is an important one since it has implications of applying optogenetics to tissue studies, in general. As far as evidence of blue light activating gap junctions, we find no evidence in the literature for this. In addition, we would argue that the small emitter PKA inhibition experiment in Figure S5C supports this. That is, in the presence of blue-light (during the second bPAC input pulse) and inhibited PKA (via H89), there is very little coupling from the emitter to the receiver as compared to the strong coupling during first pulse prior to addition of H89. A potential interpretation from these results is that if blue light somehow enhances gap junction activity, it would be through enhancing PKA activation, complementing cAMP activation. But, there is no evidence of PKA being light sensitive since we see no ERK-KTR N/C response when we illuminate all-receiver monolayer with blue light. These results suggest that gap-junction activity is not enhanced by blue light. We have added a paragraph in the Discussion for this (red text tagged with R1MC2 in Discussion, page 17 of manuscript)

The only experiment suggested by the reviewer we don't already have data for is the caged cAMP experiments in monolayers with cells that have no bPAC (receiver cells). We are assuming the reviewer wants us to illuminate a single cell (or small group of cells) to locally uncage cAMP levels, similar to our small emitter cluster experiments in the paper. However, this requires special illumination devices which we don't have, nor can we do the experiment since neither Dr. Fonseca nor Dr. Chevalier are at UCSF anymore. Furthermore, it seems that the strongest reason to do these experiments is if blue light somehow activates gap-junctions and/or enhances cell-cell coupling. But, our arguments presented above suggest that this is likely not the case. Therefore, we were hoping that the reviewer would be OK with us not doing the caged cAMP experiment.

In addition, perhaps the reviewer might want to see results of caged cAMP experiments in all monolayers for uniform illumination, i.e. all cells have cAMP uncaged. For this, we already have results for all receiver monolayers where we have added forskolin, endogenous adenylyl cyclase activator, for max endogenous production of cAMP. For this data, we observed the expected ERK-KTR response (Figure S5A from the manuscript). We realize the reviewer has already seen all this, but we are just restating it just in case it is useful.

Reviewer 1, Major comment 3: In all figures, authors exhibit traces of single cells, we regret that they do not provide more amalgamed analysis with appropriate statistics (eg time to peak, time to return to steady state, decay , ...). Also, in Fig 3 and 4, we would like to see pictures of cell status at several pertinent time points.

Authors: This is a great suggestion. In addition to the time-to-max, we have added a time-to-steady-state metric and an overshoot ratio metric.

First, the time-to-peak is very similar to time-to-max but is restricted to signals that have a peak, which can exclude signals. The time-to-max is more general which is why we use it and is still able to capture peaks due to overshoot. Importantly it also gives us an approximate time to steady-state for simple rising signals observed in all-emitter experiments, for example.

The new time-to-steady-state metric we defined this as the time the signal drops to within 30 percent of the end of pulse signal amplitude relative to the max signal amplitude, and denoted this time as *time-to-ss* in the manuscript.

The new *overshoot ratio* metric defined as $\frac{S_{max}-S_{start}}{S_{start}-S_{end}}$ where S_{max} is the peak signal during the bPAC pulse, S_{start} is signal magnitude at the start of pulse, and S_{end} is the signal magnitude at the end of pulse (see Fig 3C in revisions for illustration).

Given these two new statisical metrics, we have expanded on the original statistics analysis section that refers to Figure 3 as well as the section that refers to Figure 4. We have labeled the start of each of these sections with R1MC3, i.e. Reviewer 1, Major Comment 3, on pages 6, 8, and 10.

By cell status we assume the reviewer wants to see the the ERK-KTR distribution, nuclear and cytosolic, in cells over time such as was presented in Figure 2C (all emitter) and Figure S3B (single emitters from single emitter clusters). In our original submission, we thought it would be sufficient to show one example for the all-emitter case with the simple response (Figure 2C) as well as two examples of overshoot for the simple emitter cluster experiments (Figure S3B). The reviewer's

suggestion is very good in that it would mirror what is seen in the all emitter. We've added one more snapshot example (Figure 3B in revised manuscript) for the small emitter cluster in Figure 3A to give the reader an immediate sense of how the nuclear ERK-KTR is behaving when overshoot occurs in the ERK-KTR N/C signal. We decided not to add snapshot examples to Figure 4 because we feel it wouldn't add anything that the reader could not already visual based on the snapshot examples presented already. It also saves space in Figure 4 to make it more complete such as discussed in Comment 1 and 3 above, i.e, the extra statistical plots and the results of the small-emitter-cluster experiments with Epac and PKA inhibition.

Reviewer 1, Major comment 4: Considering cellular and intercellular cAMP diffusion velocity but also cellular resistivity between connected cells, authors should report a delay in the activation and inhibition of ERK-KTR N/C ratio between emitter and receiver at the beginning and at the end of bPAC stimulation with blue light pulse but also in the intensity of N/C ratio. Therefore, is there any distance effect in N/C intensity, delay in N/C activation/inhibition between proximal and distal receiver cells from emitter cells and a relationship between number of connected cells (cellular resistivity effect) between emitters and connected receivers. These should be mentioned. Furthermore, authors should discuss their data regarding the literature reporting cAMP diffusion in cytoplasm and between cells (eg Bock A et al Agarwal SH et al and Ponsioen B et al).

Authors: These are interesting thoughts. While the subject of this manuscript is mostly about gap-junction regulation, the propagation of the cAMP signal across cells was our first interest when we started this research project. We should note that because we ended up focusing on gap-junction regulation in single emitter clusters, we would find a single emitter cluster or two in a region of the tissue using our 40X lens, even when there might be emitters just outside the field of view. Because of this, one has to be careful about making interpretations about signals in receiver cells near the edge of the images because some could be receiving cAMP from emitters outside the field of view which contribute to their overall ERK-KTR N/C signal. This is a reason why we focused on studying the receivers adjacent to the emitter, because their ERK-KTR N/C signals are dominated by that emitter.

However, in Figure S3D (revised manuscript), we show a single emitter cluster example where the peak receiver ERK-KTR N/C amplitudes drop off as a function of distance from the emitter. This is expected when there is only one emitter in the area. In Figure S10D in the revised manuscript, for the experiment used for Figure S3D (revised manuscript), we now present the time signals of the emitter and a few receivers at different distances. Peak signal amplitude tends to decrease and time of the peak increases the further a cell is away from the emitter. As a comparison, results of our model are plotted in Figure S10C and recapitulate the trends seen in the experimental data. The model even shows a slight delay (flat before initial rise) for the cell furthest from the emitter (cell 13), as the review suggests. However, in the experiments, the further a receiver is from the emitter, the lower the amplitude is and thus the lower the signal-to-noise ratio. Thus, trying to assign delay to these receivers is not quantitative. Assigning delay in the peak of the signal is, of course, doable.

With respect to the literature, we do use the results from Agarwal SH et al (and some other theoretical insights from Hofer et al.(2001) and Nitsche et al (2004)) to support the well-mixed assumption within each cell that we use for our multicellular simulation model (see last section in S4 Text (Biophysical arguments for the well-mixed assumption)), as opposed to having to implement a full-spatial (diffusion-reaction) treatment.

Please see the paragraph in the Discussion tagged with R2SC7c and R1MC4 starting on page 18 of the revised manuscript which discusses the modeling and measurements of signal propagation (different parts of which were suggested by different reviewers). We greatly appreciate these suggestions.

For future experiments of propagation and associated delays, we would use a larger field-of-view to get more cells, at least double the dimensions. And larger emitter clusters would allow for a higher level of signal strength (higher signal-to-noise ratio) as a function of receiver distance from the emitters.

Reviewer 1, additional comment a: In fig2 legend. There is a switch between panels F and G that should be E and F.
Reviewer 1, additional comment b: In page 11, the sentence "For this case, the cAMP degradation term is simply ... Multiplied BY the rat..." and not bay.
Reviewer 1, additional comment c: Authors mentioned the used of carbonoxolone in their study, we guess it was carbenoxolone (CBX).
Reviewer 1, additional comment d: In Fig S3 legend. Panel C, authors mentioned Top and bottom, instead of left and

right.

Authors: Thank you noting these errors. We have fixed all errors associated with additional comments a-d.

Response to Reviewer 2

We greatly appreciate the time, effort, and comments by the reviewer. They have helped us improve the manuscript. We need to first address comment 7a, where their comment suggests a misunderstanding of the model by the reviewer. We take full responsibility for our lack of clarity in communicating our model in the manuscript. Upon re-reading the gap-junction model section of the main text, we realize there were vague section titles and specific details missing that would have greatly helped the reader without having to decipher the equations in the Supplemental Information. In our revisions we have taken extra care in adding important details. Again, we apologize and greatly appreciate the comments as they help make the paper much more readable. Here we discuss comment 7a:

Reviewer 2, Specific Comment 7: Model details: So I appreciated the simplicity of the model but as noted above, it is not clear what purpose was served. Some considerations that should be considered in this case:
Reviewer 2, Sub-comment 7a: If each cell is a well-mixed compartment, then why not consider a multicompartment model and use the gap junctions as flux between compartments? This would be the most logical tradeoff between a detailed spatial model and the current version of the model.

Authors: To reiterate from above, we apologize for doing a poor job of explaining the model. We are actually doing the multicompartment model with cAMP flux through gap-junctions at the cell-cell interfaces. And at the end of **S4 Text: Details of multicellular model** we apply theoretical insights from Hofer et al. (2001) [1] to justify the well-mixed assumption. It is possible that part of the misunderstanding is our title for the modeling section: *Determining a simple cell-cell coupling model*. This title makes it seem like we are developing a model of how cAMP is transferred to other cells. We are actually using a standard gap-junction flux model [1]-[2] at every cell-cell interface. We have renamed this section *Determining a simple regulated gap-junction flux model for the cell-cell coupling of cAMP*.

We will now show that our model follows a standard gap-junction flux model, e.g. [1-2], where the model for the flux, $F_{c_{ij}}$ of cAMP through gap-junctions between cell i and j is

$$F_{c_{ij}} = \rho_{ij} \times \left([cAMP^j] - [cAMP^i]\right) \tag{1}$$

where ρ_{ij} is the effective gap-junctional cAMP permeability (can be time-dependent) at the interface between cell i and cell j. And $[cAMP^j] - [cAMP^i]$ is the difference of cAMP concentrations at the gap-junction interface and dictates the direction and magnitude of the flux. We want to point out that in [1], Hofer et al. present both a full spatial treatment for a 1D multicellular system (see Eq (7)-(8) in [1])and the limiting well-mixed compartmental case (see Eq. (12) in [1] where for both cases, they have the form of Eq. (1)).

This standard model can be seen in the time-dependent cAMP equation from the minimal model in **S4 Text: Details of multicellular model**. From our original submission, this equation for the ith cell is

$$\begin{aligned}\frac{d[cAMP^i]}{dt} \;&=\; I_{bPAC}(t)\beta_{c,b} + \beta_{c,ac}\frac{X_{f_1}}{c_{ac,pk}[PKA^i_{on}] + X_{f_1}} - \gamma_c[cAMP^i] - (1 - I_{IBMX}(t))\gamma_{c,pd_b}[cAMP^i] \\ &+ \sum_{j\neq i}\omega_{ij}\left[k_{gj} + k_{gj,gjf}\frac{[GJC^i_a] + [GJC^j_a]}{[GJC^i_a] + [GJC^j_a] + X_{f_3}}\right]\left([cAMP^j] - [cAMP^i]\right)\end{aligned} \tag{2}$$

Here the cell-cell coupling through gap junctions is the term on the second line with $\left([cAMP^j] - [cAMP^i]\right)$ representing the standard difference in cAMP concentrations between the two cells. The remaining terms to the right of $\sum_{j\neq i}$ comprise ρ_{ij} from Eq (1) above. The term $\omega_{ij} = 1$ when cell i and j are coupled via gap-junctions, i.e. they interface, otherwise $\omega_{ij} = 0$. The term k_{gj} represents a basal gap-junctional cAMP permeability term and $k_{gj,gjf}\frac{[GJC^i_a]+[GJC^j_a]}{[GJC^i_a]+[GJC^j_a]+X_{f_3}}$ represents a time-varying gap-junctional cAMP permeability term that is dependent on the active gap-junction molecule concentration on the interface. Here the active gap-junction molecule concentration is a function of the activated gap-junctions initiated in both

cell i and cell j by the delayed gap-junction regulation model. While we do put in a saturation term, in all our simulations $[GJC_a^i] + [GJC_a^j] << X_{f_3}$, thus the denominator is approximately X_{f_3}. For the results in our original submission Eq. (2) is approximately behaving as

$$\frac{d[cAMP^i]}{dt} \approx I_{bPAC}(t)\beta_{c,b} + \beta_{c,ac}\frac{X_{f_1}}{c_{ac,pk}[PKA_{on}^i] + X_{f_1}} - \gamma_c[cAMP^i] - (1 - I_{IBMX}(t))\gamma_{c,pd_b}[cAMP^i] + \sum_{j\neq i}\omega_{ij}\left[k_{gj} + \frac{k_{gj,gjf}}{X_{f_3}}\left([GJC_a^i] + [GJC_a^j]\right)\right]\left([cAMP^j] - [cAMP^i]\right) \tag{3}$$

For the revision, we have adjusted the model to be similar to Eq. (3) but with more explicit cell-cell interface information as follows

$$\frac{d[cAMP^i]}{dt} = I_{bPAC}(t)\beta_{c,b} + \beta_{c,ac}\frac{X_{f_1}}{c_{ac,pk}[PKA_{on}^i] + X_{f_1}} - \gamma_c[cAMP^i] - (1 - I_{IBMX}(t))\gamma_{c,pd_b}[cAMP^i] + \sum_{j\neq i}\omega_{ij}\left[k_{gj} + \frac{k_{gj,gjf}\bar{P}}{X_{f_3}}\left(\frac{[GJC_a^i]}{P_i} + \frac{[GJC_a^j]}{P_j}\right)\right]\left([cAMP^j] - [cAMP^i]\right) \tag{4}$$

where P_i is the number of cells that cell i couples to and P_j is the number of cells that cell j couples to. And as discussed above $[GJC_a^i]$ is the active concentration initiated in cell i and likewise $[GJC_a^j]$ in cell j. Within each cell, the active concentration is parsed equally to each cell they interact with, hence $\frac{[GJC_a^i]}{P_i} + \frac{[GJC_a^j]}{P_j}$ are the parsed concentrations from cells i and j, on the ij interface. The term $\bar{P}$ is the average number of cells that the cells couple to. In this work we set $\bar{P} = 5$, where most P_i typically vary between 4 and 7. Thus when $\bar{P} \approx P_i \approx P_j$, then one can see that Eq. 4 has the same form as Eq. 3 since the $\bar{P}$ cancels the P_j and P_j. The numerical model results using Eq. (4) are very similar to those using either Eq. (3) or Eq. (2) (Figure 1 in this response, single emitter cluster experiment results).

It is obvious we need to do a better job of introducing and discussing the gap-junction flux model and how it is regulated by the delay gap-junction regulation model. To address this, we have put more of the model description and assumptions in the main text including explicitly stating that the delay gap-junction regulation model is modulating the effective gap-junctional cAMP permeability, ρ_{ij}. For any single-emitter-cluster simulation in the paper that used Eq. (2), we have re-simulated them with Eq. (4). The updates in the main text of the manuscript are in blue and tagged with R2SC7a, on pages 11 and 13 of the manuscript. And we have updated the equations and text in S4 Text (Details of multicellular model) as well, wherever it was necessary.

1 J. M. Nitsche, H. C. Chang, P. A. Weber, and B. J. Nicholson. A transient diffusion model yields unitary gap junctional permeabilities from images of cell-to-cell fluorescent dye transfer between Xenopus oocytes. Biophys J, 86(4):2058-2077, Apr 2004.

2 T. Hofer, A. Politi, and R. Heinrich. Intercellular Ca2+ wave propagation through gap- junctional Ca2+ diffusion: a theoretical study. Biophys. J., 80(1):75-87, Jan 2001.

Reviewer 2: Review Comments to the Authors: I read this paper with great interest and found both the experimental details and the questions fascinating. Since I'm not an experimentalist, I will leave it to the other reviewers to comment on the various controls. However, I have a fundamental question – what is the purpose of the modeling? I don't mean this to be disrespectful of the authors efforts but I think the text doesn't clarify why a model is necessary and what is to be gained by this additional effort. Since the experiments are so well-contained and the manuscript as written doesn't necessarily test any predictions that were made from the model, it could be interpreted as an exercise in curve-fitting (to be clear, I'm not saying that it is). I'm wondering if there is a missed opportunity here for the authors to make the case that the model clarifies something that is not experimentally obvious or feasible.

Authors: We are very happy the reviewer enjoyed learning about the experiments and the questions that inspired them. The data measured from the experiments in the first half of the paper implicates gap-junctions as the mechanism for the overshoot observed in the ERK-KTR N/C signal in single emitters from single-emitter-cluster experiments. However, our experiments do not measure the gap-junction activity at cell-cell interfaces. That is where the model comes in to play.

Given that our multicellular model calculates the time-dependent active gap-junction concentrations at all cell interfaces, we are therefore calculating all time-dependent cAMP fluxes between cells. Very importantly, this model allows us to test different time-dependent gap-junction regulation models on the active gap-junction population at each cell-cell coupling interface. We initially show that constant gap junction activity at cell-cell coupling interfaces cannot reproduce the overshoot seen in the ERK-KTR N/C signal in single emitters within a single-emitter cluster experiment. This lead us to develop the delayed gap-junction regulation model that drives the time-dependent gap-junction state (effective gap-junctional cAMP permeability) at each cell-cell interface. This is incredibly important because the model is determining the time-dependent physical state of the interface that is responsible for the flux of cAMP between cells.

And we do make model predictions followed up by experiments (discussed in main text section *Model predictions and experimental validation* with results presented in Figure S8). The cases we present are those that we had the resources and expertise to follow through on.

The reviewer has made us realize the importance of presenting the time-dependent gap-junction state, especially at the emitter/receiver interface. In the original submission we had placed two examples in a supplemental figure. However for the revision, for Figure 5I we now include a plot of the time-dependent gap-junction state. And for the example in Figure S8A and those in Figure 6, we present plots of the corresponding gap-junction state in Figure S9. The updates in the main text of the manuscript that reference these plots are in blue and tagged with R2OC, on pages 13, 16, and 17 of the manuscript.

Reviewer 2: Specific Comment 1: Time dependent signal varies from cell to cell – but do you separate signals that arise from gap junction-mediated cAMP versus cell made cAMP?

Authors: Except for one specific experiment which we'll discuss below, we aren't able explicitly separate them. We just show that the emitters in all emitter tissues have a very simple ERK-KTR N/C signal while single emitters can have dramatic overshoot. This is why the model is important. Having a simple intracellular circuit model that captures the simple intracellular ERK-KTR N/C signal, we are able to then test gap-junction regulation models that can yield the type of signals we observe.

The special experiments is from Figure S5C (original submission (or Figure 4C revised manuscript)), where the emitter exhibits quick overshoot in the ERK-KTR N/C signal during the first pulse. Halfway between the end of the first pulse and start of the second we add PKA inhibitor (H89) which inhibits cell-cell coupling of cAMP. We then observe a simple first order response typical of the all-emitter tissues, but reduced in amplitude since loss of PKA activity reduces the amplitude of ERK-KTR N/C response, but has little effect on the time-dependent behavior (see Figure S5B). And since the ERK-KTR N/C signal is quite repeatable across pulses, we can infer that the signal during the first pulse due to the sum of both effects. And the signal during the second pulse is purely intracellular whose amplitude is reduced due to PKA inhibition. It's not perfect but it is a start. Finding clean gap-junction inhibitors that are fast and don't affect PKA, Epac, or cAMP degradation through PDEs would be the next step.

We appreciate the question as it is something we have thought about. And our model which we developed before this experiment ever happened, predicted this result.

Reviewer 2: Specific Comment 2: What about growth hormones from the culture medium? What about the naturally present cyclase activation? Are these not important?

For growth hormones we grow the cells using standard MDCK media with about a 24 hour cell division rate. However, for imaging, the growth hormones are reduced by a factor of 10 to reduce the cell division rate, but it cells still occasionally divide during experiments that are 3-4 hours long. The concentration of the hormones are at a constant level over the experiment, thus, EGFR signaling through ERK will not change much. This is observed in most cells where the ERK-KTR N/C signal mains a relatively constant baseline. Some cells exhibit an EKR baseline that changes slowly over time, but typically much slower than their ERK-KTR response to bPAC.

The endogenous adenylyl cyclase (AC) are still active but at much lower level. We do Forskolin (AC activitator) experiments to measure max AC activity which shows similar ERK-KTR response to lower amplitude bPAC excitation in the paper (Figure S5A). Importantly, we are not activating GPCRs which would activate AC. But there are known feedbacks on

AC that we do model. We also model basal AC activity to give the cells basal levels of cAMP.

Reviewer 2, Specific Comment 3: Time scale of the stimulus? Seems rather long – 40 min? Why was this chosen? What are the implications of this timescales of cellular health?

Authors: Those are good questions. We've done 8 hour experiments on single tissues, 40 min on, 40 minute off, and the cells are fine. The 40 minutes was chosen because the majority of cells reach steady state by that time. Twenty minute pulses are too short where the ERK-KTR N/C signal can still be exhibiting dynamics at 20 minutes. In Materials and Methods, we have added a 'Choice of input pulses' section (blue text tagged with R2SC3, on page 19 of manuscript) that discusses this choice.

Reviewer 2, Specific Comment 4: There is no figure 2G marked.

Thank you, we slightly altered Figure 2 right before submission and didn't properly alter some of the text. Our apologies. We have fixed it.

Reviewer 2: Specific Comment 5: How are the ratios of emitters to receivers controlled? Is it possible to control them?

Authors: For single-emitter-cluster experiments, we typically mix 10 percent emitters and 90 percent receivers, let them settle overnight to form a monolayer, and then peruse through the tissue using with the microscope to find single emitter clusters. Even with this approach, there can be large emitter clusters (10 or more). This is because mixing introduces some randomness, so while there are single emitter clusters, there can also be larger emitter clusters. And it is also possible that some groups of emitters never break up into single emitters and thus stay that way within the emitter/receiver monolayer.

Reviewer 2: Specific Comment 6: Figure 3: Hard to make the case for any strong correlation between single cell emitters and their neighbors. Is there critical number of cells needed for a cluster to be functional?

Authors: We are uncertain what the reviewer means by correlation. All the single emitters respond to bPAC, as well as adjacent receivers. Some respond more strongly than others, but generally their ERK-KTR N/C signals go up during the input pulse and then go down when the pulse turns off. We also don't understand what the reviewer means by 'functional'.

Reviewer 2, Specific Comment 7: Model details: So I appreciated the simplicity of the model but as noted above, it is not clear what purpose was served. Some considerations that should be considered in this case:
Reviewer 2, Sub-comment 7a: If each cell is a well-mixed compartment, then why not consider a multicompartment model and use the gap junctions as flux between compartments? This would be the most logical tradeoff between a detailed spatial model and the current version of the model.

Authors: We addressed comment 7a at the beginning of the response above.

Reviewer 2, Sub-comment 7b: What about the role of noise? Noise would be inherent in these cellular phenomena and the role of extracellular and intracellular noise should be discussed and somehow incorporated in the models.

This is a interesting question. Regarding extracellular noise, does the reviewer mean **extrinsic** noise (cell-cell variability in things such as protein expression, metabolic state, or transcriptional state, for example)? And by intracellular noise do they mean **intrinsic** noise (e.g., stochastic pathway signal fluctuations for given expression levels of pathway components)?

As for a full stochastic treatment of the system, that is beyond the scope of this paper and we feel is unnecessary. While one could model intrinsic noise, it will likely not show much beyond a noisy version of what the deterministic equations produce. Some important cases where intrinsic noise can play an important role is when there is a threshold dependent positive feedback that can amplify fluctuations and put the cell in a different state or produce fluctuation induced pulses. A sequence of double inhibitions (and mutual inhibitions) can also act as an effective positive feedback and produce similar results. For these systems, it can be shown that for certain parameter regimes, the deterministic equations cannot capture these effects produced by the stochastic system. However, our system is simple and cAMP is getting degraded by PDEs and transported through cell-cell coupling. And there is no way to amplify the cAMP signal as it is controlled by us, thus there will be no fluctuation induced amplifications of cAMP. From this, we argue that the deterministic case is sufficient, especially with respect to **intrinsic** noise.

However, we can still incorporate **extrinsic** noise into our deterministic model, by varying pathway protein levels across cells. Given that we have already done a lot modeling in the paper, we have kept it simple by varying only bPAC expression. For modeling all-emitter experiments, the bPAC expression is varied across the cells. This allows for heterogeneity in emitter ERK-KTR N/C signals. In addition, for modeling single-emitter-cluster experiments, we vary the bPAC expression in the emitter across different experiments while keeping everything else the same. For these cases, overshoot in the emitter's ERK-KTR experiments is maintained across experiments. For the presentation of this new modeling in the manuscript, please see the paragraph in the Discussion tagged with R2SC7b on page 17 of the revised manuscript. We appreciate the reviewer's thoughts to help make the modeling more thorough.

Reviewer 2, Sub-comment 7c: I think given the imaging that the authors have, a rudimentary spatial model would allow for more insight into the diffusion of signals (after all, the gap junctions are boundary fluxes then) rather than the current formulation.

Authors response: Given that our model is a well-mixed compartmental model with gap-junction coupling, we already have these results. Please see the paragraph in the Discussion tagged with R2SC7c and R1MC4 on page 18 of the revised manuscript which discusses the modeling and measurements of signal propagation (different parts of which were suggested by different reviewers). The paragraph also refers to Figure S10C-D in the revised manuscript. We we greatly appreciate these suggestions.

Reviewer 2, Stylistic comment: intro can be shortened to focus on the main problem. (This is obviously each person's preference but I'm sharing in case it is useful)

We appreciate the comments. We are happy with the length of the intro.

Reviewer 2, Minor comment: Check formatting of quotation signs and subscripts for calcium, ip3 etc. The use of the word tissue is misleading perhaps. Suggest that the authors stick with monolayers?

Great suggestion. We have updated the manuscript with only the word monolayer when discussing our results.

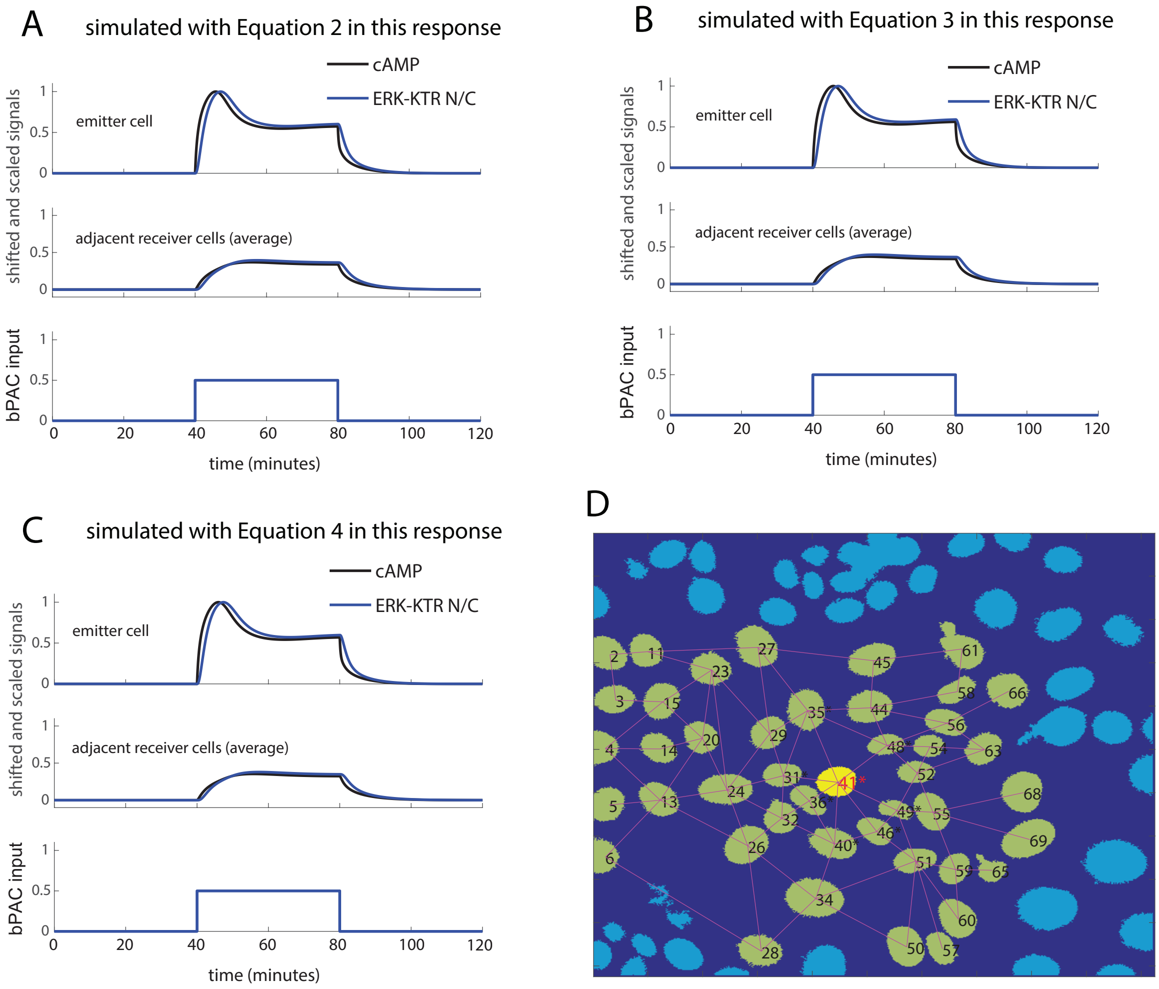

Figure 1: **Multicellular model with delay gap-junction regulation model applied to a single emitter cluster experiment.** (A-C) For top two plots, ERK-KTR N/C and cAMP signals shifted to zero and scaled for comparison, with the peak of the emitter signal at 1 and minimum at zero. The receiver signals are multiplied by the same scaling factor as the emitters to maintain the same relative amplitudes (proportionally). (A) Model simulated with Eq. (2) in this response for the multicellular system in (D). (B) Model simulated with Eq. (3) in this response for the multicellular system in (D). (C) Model simulated with Eq. (4) in this response for the multicellular system in (D). (D) Multicellular system to be modeled with 1 emitter (red label, yellow nuclei) and receiver cells (light green nuclei). Lines connecting nuclei represent direct cell-cell cAMP coupling through gap-junctions.
